# GloMPO (Globally Managed Parallel Optimization): a tool for expensive, black-box optimizations, application to ReaxFF reparameterizations

**DOI:** 10.1186/s13321-022-00581-z

**Published:** 2022-02-16

**Authors:** Michael Freitas Gustavo, Toon Verstraelen

**Affiliations:** 1 Center for Molecular Modeling, Ghent University, Ghent, Belgium; 2 Software for Chemistry and Materials, De Boelelaan 1083, 1081 HV Amsterdam, The Netherlands

**Keywords:** ReaxFF, Global optimization, Reparameterization, Black-box optimization, Python, Parallel computation

## Abstract

In this work we explore the properties which make many real-life global optimization problems extremely difficult to handle, and some of the common techniques used in literature to address them. We then introduce a general optimization management tool called GloMPO (Globally Managed Parallel Optimization) to help address some of the challenges faced by practitioners. GloMPO manages and shares information between traditional optimization algorithms run in parallel. We hope that GloMPO will be a flexible framework which allows for customization and hybridization of various optimization ideas, while also providing a substitute for human interventions and decisions which are a common feature of optimization processes of hard problems. GloMPO is shown to produce lower minima than traditional optimization approaches on global optimization test functions, the Lennard-Jones cluster problem, and ReaxFF reparameterizations. The novel feature of forced optimizer termination was shown to find better minima than normal optimization. GloMPO is also shown to provide qualitative benefits such a identifying degenerate minima, and providing a standardized interface and workflow manager.

**Supplementary Information:**

The online version contains supplementary material available at 10.1186/s13321-022-00581-z.

## Introduction

### High-dimensional, expensive, black-box optimization

In this work, we are particularly interested in tackling the hardest of global optimization challenges: high-dimensional, expensive, and black-box (HEB) problems [57]. Many real-life applications fall into this class. Black-box optimization problems—ones for which no gradient information is available—are generally regarded as some of the most difficult to handle. This is because optimizers can easily be led astray by rough surfaces, and many more function evaluations are typically needed for the optimizer to learn about the structure of the problem. High dimensionality also demands increased function evaluations, but a high evaluation expense makes this infeasible. The consequence of this complexity is a significant reduction in the number of optimization algorithms which can be used. Numerous options exist to tackle problems with one or two of these difficulties, but rarely are all three addressed simultaneously [57].

A particular reason for our interest in HEB problems is the practical challenges they introduce to the optimization process. A practitioner faced with a new optimization challenge must select an algorithm, and then values for its hyper-parameters. These choices are made based on some intuition of the problem, but are often shown to be wrong as the task is investigated further. Often, optimizations become iterative procedures to refine algorithms and their settings, and to verify the quality and reproducibility of the minima found. When the task is both difficult and expensive, this procedure can become time-consuming and difficult.

### Metaheuristics

Tackling these hard problems can only be done with metaheuristics, i.e., the use of a two-tier algorithm. A metaheuristic is any optimization method in which an upper algorithm selects the starting conditions for a lower one. The lower level is typically any local search procedure and is called a heuristic. The heuristic may provide a suitable solution to the problem but cannot be used alone since it will most likely locate a local rather than global minimum [5]. The use of metaheuristics decouples, and attempts to balance, exploration and exploitation.

Metaheuristics is a very broad term. The literature on it is extensive and varied, and the term is often not used explicitly. Nevertheless, most optimizers are metaheuristics. This includes solution-based methods (which iteratively improve a single incumbent solution), and population-based ones (like evolutionary algorithms (EA) which improve a group of solutions). A similar, but less broad, term which is also encountered is ‘multi-start’ optimization which refers to the repeated application of local optimization steps [42].

It is not always obvious that an algorithm applies this two-level structure. For example, the simulated annealing technique uses a ‘temperature’ parameter to govern how far the optimizer can look from the incumbent solution. The temperature is decreased during the optimization to slowly focus the optimizer on a minimum; this represents the upper method. The lower-level heuristic is simply a function evaluation, but in the Python SciPy [66] implementation of an annealing strategy this has been replaced with a local search algorithm.

Another simple example is the efficient and popular basin-hopping (BH) strategy of Wales and Doye [67] which couples a specially configured sampling strategy with local optimizations.

Both of the above examples use Monte Carlo steps as the metaheuristic to govern local search locations. An important advance to this came in the form of tabu search [20] which introduced ‘memory’—the concept of previously visited points influencing subsequent steps.

In the case of population-based metaheuristics the upper algorithm is the crossover and mutation of individuals, and the heuristic is evaluations or local searches. Many novel ideas within the realm of EAs have stretched the study of metaheuristics further. One such technique is the use of subpopulations. This term has been used to refer to the splitting of a problem into a collection of subproblems that are solved simultaneously [70, 71], or, more commonly, the use of multiple populations to solve a problem [10, 50, 53]. The latter technique is used to maintain diversity, and some algorithms allow for information to be shared between the populations through a process called ‘migration’ [1, 21]. ‘Niching’ is another technique used by EAs to maintain population diversity by ensuring the population has at most one member (or a small number) in any given niche. Niching may be as simple as discretizing the search space [43, 58] or introducing some other measure of difference between individuals [28, 46, 68]; the latter choice overlaps with the concept of ‘order parameters’.

Order parameters are some measure, other than the inputs and outputs of the optimization problem, by which solutions are ranked or choices are made by the meta-algorithm. These are perhaps the most powerful aids to solving HEB problems because they introduce extra problem-specific information to the optimizer. If not selected carefully, they run the risk of biasing the algorithm, and are generally not transferable to other types of problems. However, the use of order parameter has been repeatedly shown to dramatically improve performance by maintaining diversity and reducing the enormous search space of high-dimensional problems [7, 9, 10, 28].

One technique which is ubiquitous in literature is ‘hybridization’—the act of coupling existing lower- and upper-level algorithms in new ways [7, 23, 49, 50, 68]. Over the years, a plethora of exploration and exploitation algorithms have been applied in every combination. Typically, they introduce some improvement, however, the number of publications of this type has generated some criticism [59]. We make the observation that, unfortunately, many algorithms are published without making an implementation available.

### Optimizer supervision

In more recent years, in order to take advantage of the advances in computational infrastructure, efforts have been made to parallelize optimization algorithms. The result is usually a parallel exploitation step (which simultaneously explores several basins), coupled to a single serial global exploration step. In this way, full parallelism is not achieved. A particularly interesting extension of these efforts, which seems to have gotten little attention in the literature, is the concept of using the metaheuristic to monitor the performance of the lower-level heuristic.

Schutte et al. [54] tackled the problems of efficient parallelism, and convergence to local minima, in a novel multi-start approach. In their work, optimizers are characterized as exploring for a certain number of iterations, and then converging to a point and spending many iterations fully focused on that area. Their algorithm assumes that instances of an optimizer will all spend about the same number of iterations exploring a domain before focusing. Thus, a single optimizer is run, its ‘exploration’ time measured, and then several new optimizers are spawned and allowed to run for the same period of time. Through this approach the authors were ultimately able to prove that multiple independent optimizations improved the probability of global convergence. Rather than setting the maximum number of function evaluations *a priori*, Swersky et al. [61] devised a Bayesian-based termination condition to stop optimizers which had reached convergence. Although the idea shows promise, we found this approach to be overly complex and computationally expensive during our own testing. Yang et al. [70, 71], in their extended CCFR and CCFR2 frameworks, dynamically allocated computational resources to their subpopulations based on performance.

From these works we note, generally, that optimizers tend to converge to local minima, and spend several hundred iterations exploring regions that should perhaps be abandoned because a more promising region exists elsewhere. If multiple optimizers were run simultaneously, and compared in real-time, these convergences to poor minima could be immediately identified and stopped.

### Globally managed parallel optimization

Bearing in mind the difficulties associated with HEB optimization, and adapting elements from the previously mentioned sources, we present a novel optimization framework called GloMPO (Globally Managed Parallel Optimization). GloMPO is a customizable metaheuristic that explicitly splits the algorithm levels. The upper level functions as an optimization manager that launches, controls, and supervises parallel executions of the lower-level heuristic. The manager is designed to stand atop and outside of traditional optimization algorithms, providing real-time supervision, control, and information sharing.

Unique to GloMPO is its ability to act as a supervisor and to terminate its subordinates early. This guarantees the efficient use of both computational resources, and the evaluation budget, while simultaneously increasing the probability of finding better solutions through its multi-start approach as proven in Schutte et al. [54].

GloMPO allows one to deal with many of the problems mentioned above, while incorporating many of the best ideas:

1. By running optimizations in parallel (potentially using different algorithms and settings), allowing for automated termination, and providing opportunities for real-time interventions by users, GloMPO deals with a lot of the practical optimization challenges mentioned at the end of “High dimensional, expensive, black-box optimization”.
2. We believe GloMPO is the first metaheuristic to formalize an active supervision and termination mechanism in the way it is implemented here.
3. GloMPO is constructed in an object orientated manner such that all decision criteria can be pieced together with simple code stubs. This makes the framework totally customizable, and allows users to hybridize and mix new optimizations together.
4. As GloMPO grows, its library of components will increase. This allows code to be reused efficiently. Many algorithms in literature are not published with a publicly available implementation. This limits their usage to those users who are prepared to reimplement the idea themselves. A framework like GloMPO allows these algorithms to be mimicked with existing code.
5. The GloMPO manager provides a centralized and efficient logging mechanism of points visited by its children. This allows its decision-making machinery to make memory-based choices like tabu search.
6. GloMPO runs optimizations totally independently and in parallel which maximizes computational resource efficiency.
7. GloMPO allows functions to return any extra information they like. These can be used as order parameters by decision criteria to further guide the search.

In this way GloMPO touches on all of the most important points mentioned in the literature review above in a single user-friendly framework.

Several metaheuristic optimization frameworks (MOF) have already been developed. Implementations were found in both Java [12, 13, 36, 41, 47] and C++/C# [14, 15, 18, 34]. Unfortunately, some of these existing frameworks are commercial [44] and others are no longer in development [12, 13, 15, 45]. With few exceptions, the MOFs we located are strictly evolutionary algorithms which allow users to mix and match operators and selection functions.

We did not find a MOF implemented in Python. We believe this would be a valuable contribution given the language’s current and growing popularity in the data science space [63]. GloMPO is also more versatile than most implementations since it is not limited to EAs, and, as far as we can tell, is unique in presenting a supervision structure with forced termination of parallel searches.

The remainder of this paper is dedicated to demonstrating GloMPO’s abilities on a variety of optimization problems. Some algorithmic details are included in “Implementation” and “Methods” documents the benchmarking procedure. “Results and discussion” details three tests performed through the framework to demonstrate: (1) the advantages of its management abilities, (2) its ability to mimic other optimization algorithms by piecing together various strategies, and (3) its assistance on real-life optimization problems in which human interventions are often needed to guide the process.

## Implementation

### General structure

**Fig. 1:**
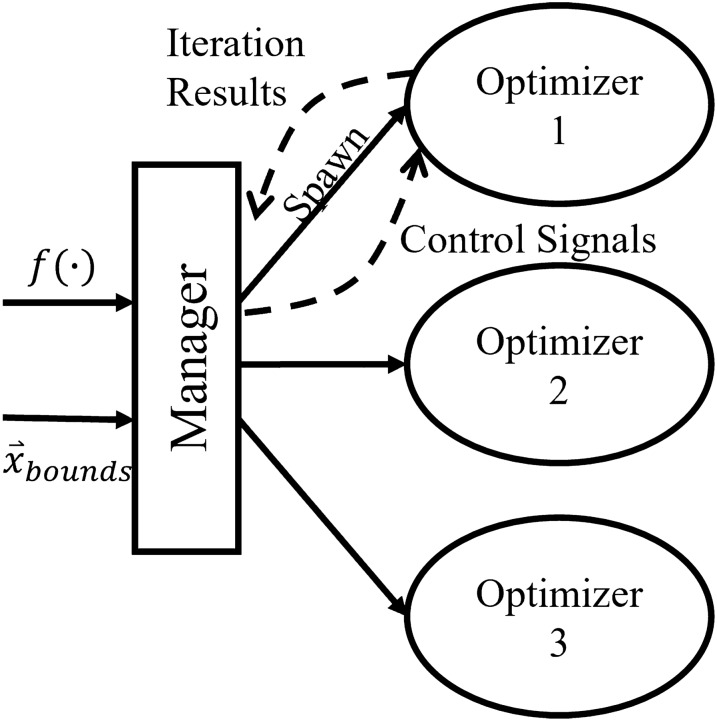
Schematic representation of the GloMPO premise

GloMPO is a metaheuristic with two algorithmic levels, and one is subordinate to the other. A schematic of the GloMPO structure is given in Fig. 1. The upper level or ‘manager’ is responsible for monitoring, controlling, and sharing information between structures of the lower level (the ‘children’). The children are traditional optimization algorithms which attempt to locate a minimum of the cost function. Depending on one’s strategy, children can be global or local search algorithms, i.e., GloMPO can work as a metaheuristic, or act at an even higher level.

**Fig. 2:**
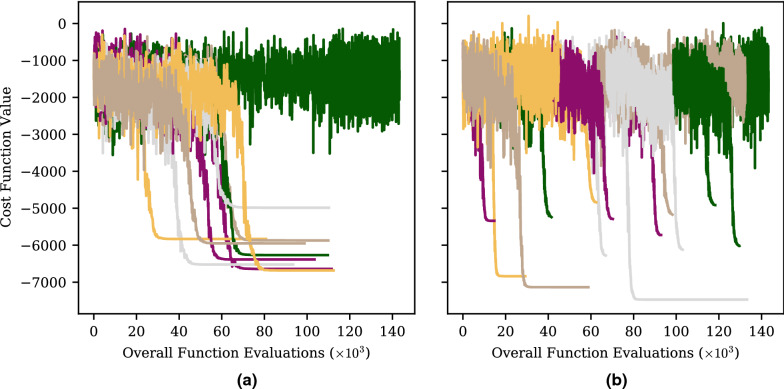
Comparison of (**a**) unmanaged and **(b)** GloMPO managed optimization of 20D Schwefel function using CMA-ES optimizers

The rational for the supervision and control mechanism is best demonstrated by illustration. An example of typical optimizer behavior is given Fig. 2a. This shows the objective function evaluations of ten CMA-ES optimizers over time. The optimizers work independently, but were all given the same optimization function; 20D Schwefel (see “Optimization task”). Each optimizer was started at a random location. For clarity, the x-axis refers to the cumulative number of function calls used by all optimizers. In the case of CMA-ES, each optimizer iteration involves several function evaluations, only the best evaluation of each iteration is shown in the figure.

Most optimizers spend some period of time searching quite globally, and not substantially improving their incumbent best solution. At some point, the optimizer will rapidly converge towards a single value, shrink its exploration radius, and spend a large number of iterations on marginal improvements until a desired tolerance is achieved. It is very rare for substantial progress to be made once an optimizer is within this ‘focus’ phase. It follows then, that focusing can represent a significant waste of function evaluations if another child is simultaneously exploring a better region. Some optimizers never reach this focus phase and continue to explore without convergence for hundreds of iterations.

Both of these behaviors represent inefficiencies in the use of function evaluations. By monitoring such optimizers in real-time, the manager is able to step-in and terminate poor performing children, and start new ones in their stead. This replaces human interventions which are typically required during HEB optimizations.

Figure 2b shows an example of what managed optimizer trajectories look like. In this figure, the same 20D Schwefel function was used. The number of function evaluations was limited to the same number used by the unmanaged optimizers. Within this same limit, more optimizers are started, more minima are identified, and optimizers are only allowed to focus on the deepest of them. As a consequence, a lower overall minimum is found.

In this way GloMPO aims to use the available iteration budget more efficiently. A second important attribute of this approach is the ability to use information from earlier children to improve the starting position and configurations of future children. In fact, information can be shared between optimizers during the optimization. The benefits of these are explored fully in “Results and discussion”.

### Python implementation

**Fig. 3:**
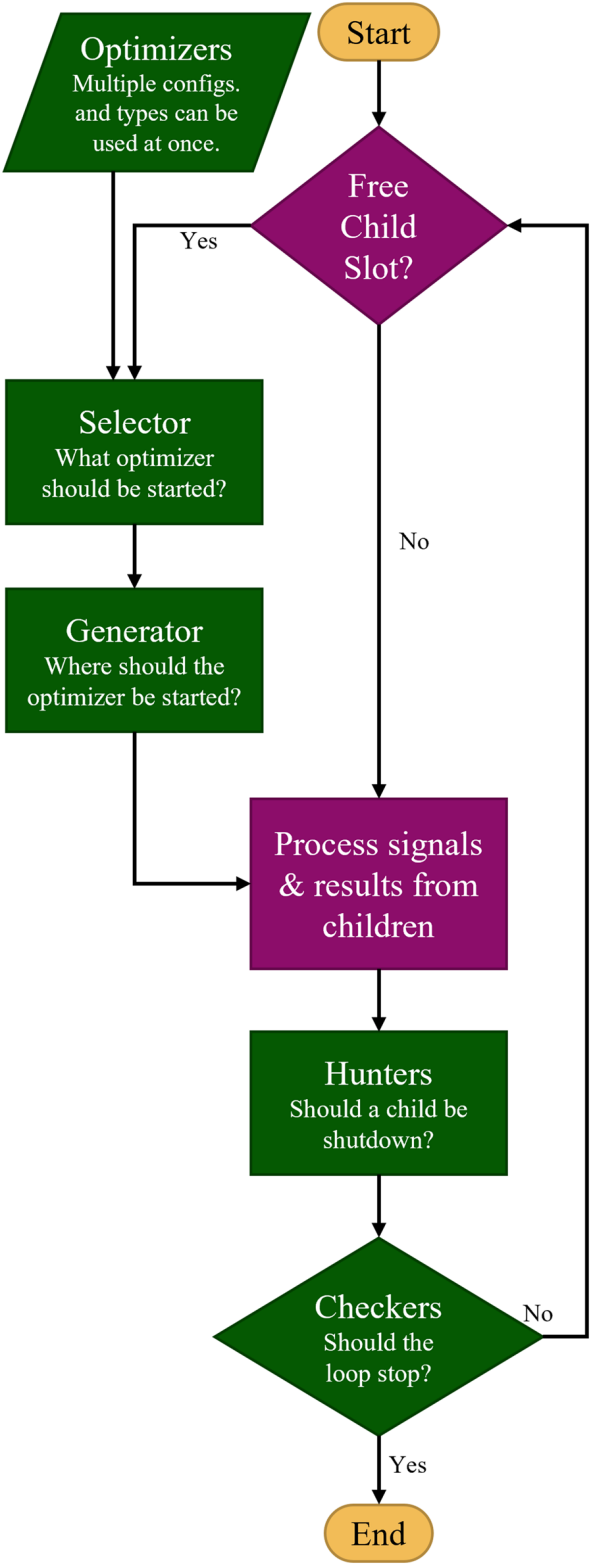
Simplified decision tree of GloMPO manager loop with customizable modular code stubs colored in green

GloMPO has been implemented in an open-source Python package [16]. For ease of use, and to allow for customization by users of all programming strengths, a plug-and-play approach has been chosen for each decision criterion. In this way an optimization can be configured by a collection of small, easy-to-write code stubs. GloMPO comes bundled with several of the most common and basic classes, but the user is free to implement their own. Figure 3 shows a simplified workflow of the manager control loop, with the user-customizable code stubs colored in green. In total, the five classes allow for a great level of customization and can be used to construct sophisticated workflows. They can also be extremely straightforward for ease of use. The choice depends entirely on the difficulty of the task, and the user’s insight. Each of the five types of customizable classes are detailed below:

- *Optimizers* GloMPO can use any existing optimization algorithm as one of its children. Wrappers already exist for CMA-ES [26] and Facebook’s Nevergrad [48], which is itself a wrapper around most common algorithms. This gives GloMPO greater flexibility than some MOFs in literature which are limited to EAs.
- *Selectors* Selectors chose which optimizer to start from an available pool of configurations. GloMPO is able to manage different types of children at once. This allows the manager to start a certain type of algorithm early in the optimization and replace them with another type later. The selection can also be based on feedback from other children.
- *Generators* These functions provide starting locations for new optimizers. One type of generator could provide random points, while another might base its choice on promising regions of the domain seen by existing children, a third might use a Latin hypercube sampling approach to ensure that the children are adequately distributed throughout the space. Generators act as the upper algorithm of metaheuristics.
- *Hunters* The decision criteria for terminating an optimizer early are provided by hunter objects. These are simple code stubs which GloMPO allows to be combined together using logical statements to create sophisticated and specialized termination conditions. For example, one might begin an optimization with one type of optimizer to quickly identify basins of interest, terminate these, and then begin a second type of local optimizer to explore these basins and terminate them if they converge to higher values than another child.
- *Checkers* Similar to hunters, these conditions can also be combined to control when the manager as a whole stops its routine. This can be based on computation time, number of iterations used, reaching a target function value, converging a number of children, or any other such condition.

GloMPO supports parallelism at two levels: (1) the manager parallelizes the optimizers, (2) the optimizers may parallelize the function evaluations (if the optimizer algorithm supports this). Both levels can be threaded and run as processes. The choice depends on the evaluation speed of the objective function as well as interfaces to external software (i.e., whether they are thread/multiprocess-safe or not). GloMPO does not currently support parallelism over multiple nodes, however, it is a feature we are interested in implementing in the future. For a more detailed explanation of the implementation, please see the Additional file 1: Section S2.

## Methods

As a framework, GloMPO’s scope is enormous. There is much to uncover in terms of optimal configurations, how to make hunting more intelligent, which tasks or optimizers are suited to management, etc. As an introductory paper, we have limited ourselves to three goals, and leave other questions unanswered for further investigation.

In the following sections, we will demonstrate the following:

A Active supervision and forced termination (hunting) of parallel optimizers can make use of an evaluation budget more efficiently and locate better minima;
B GloMPO can be used to mimic and outperform other metaheuristic algorithms;
C GloMPO can aid users in finding better minima for extremely hard, real-life, minimization problems; namely, the reparameterization of ReaxFF force fields.

### Benchmark test procedure

To demonstrate GloMPO’s effect, in each of the three tests above, it must be shown that GloMPO is statistically more likely to find lower minima. This is done by comparing the final results of unmanaged and managed optimization schemes when given the same task and evaluation budget, and repeated several times. In the context of this work, we refer to the unmanaged optimizations as ‘serial’ optimizations. This is in reference to: (1) metaheuristic algorithms, like basin-hopping and dual annealing (DA), which serially apply local optimizations, and (2) the typical approach when dealing with difficult problems of repeating optimizations several times to offset the risk of converging to a local minimum.

Algorithm 1 details the benchmark test devised to fairly compare serial and GloMPO optimizations. In it $n_s$ optimizers are run serially, and each is allowed to converge naturally without limits on time or number of iterations used; in other words, they search until a function tolerance or other internal convergence criteria is reached. The sum of all function evaluations used by each serial optimizer (*serial_evals_used*) forms the budget for the GloMPO competitor. A serial/GloMPO pair linked in this way through the evaluation budget is referred to here as a ‘bout’. GloMPO is given the same optimization task ($f(\cdot )$) and uses the same child optimizers as in the serial run, in this way any difference in performance is directly attributable to the management aspects of GloMPO. GloMPO manages $n_g$ optimizers at once; each of these may be shut down and replaced at any time, but the total alive at any one moment is $n_g$. The winner of the bout is the optimization approach which achieves the lowest answer. A total of $n_b$ bouts are performed for statistical significance.

We note that the results of the benchmark test are a function of the hunting paradigm and the optimizer(s) used. Of course, these two aspects are also further correlated; hunting works on optimizers differently, and their combined behavior depends on the minimization task. It would be beyond the scope of a single paper to investigate every possible combination of optimizer, task, hunter, etc. Thus, these results should not be inferred to apply generally, but rather to be indicative of the advantages that are possible. 
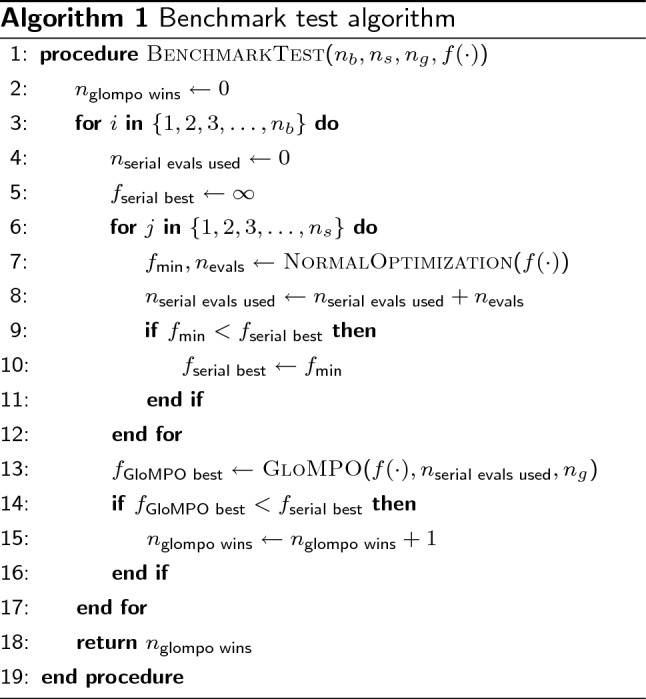


### Hunters

Four hunter conditions, detailed in Table 1, are used in this work. The basic hunter template was:

$$\begin{aligned} & \quad ({\texttt {EvaluationsUnmoving}} \\ & \mathbf{and} \quad {\texttt {ValueAnnealing}}) \\ & \mathbf{or} \quad {\texttt {BestUnmoving}} \\ & \mathbf{or} \quad {\texttt {ParameterDistance}}. \end{aligned}$$

The parameter values used for the hunters depended on the function being optimized, and were selected *ad hoc*. The ValueAnnealing and ParameterDistance hunters were not used in tests using N-CMA (see “Child optimizers”).

**Table 1:** Description of the types of hunters used in the benchmark tests

Hunter class	Description
EvaluationsUnmoving (call, tol)	Calculates the standard deviation of the last calls function evaluations. Returns true if this value is smaller than tol times the last function evaluation. Used to terminate an optimizer when its function evaluations are unchanging, i.e., when it has reached its focus phase.
ValueAnnealing (med_kill_chance)	The probability of returning a kill signal follows an exponential distribution based on the difference in function value between two optimizers. The chance of killing an optimizer twice as large as the lowest optimizer is med_kill_chance. Optimizers which are exploring values which are close to one another are less likely to be killed than those far apart. Used as a way to save optimizers which are competitive and may become the best.
BestUnmoving (calls, tol)	Kills an optimizer if it has not improved its best ever function evaluation by at least tol percent in calls. Used to terminate optimizers that explore for too long without focusing on to a point.
ParameterDistance (relative_tolerance)	Kills optimizers which are exploring points in the domain which are separated by a distance less than relative_tolerance times the maximum distance between any two points within the bounded domain. Used to terminate optimizers in the same basin.

The hunting configurations used in this paper represent common-sense empirical termination condition which one might employ when optimizing a new function about which little is known. In our testing we found that the hunter configuration was very important to GloMPO’s performance. The development of a more rigorous hunting framework would be an important next step in development. Readers can consult the results files for detailed hunting configurations.

### Test A: advantages of management

The purpose of the first experiment, Test A, is to investigate the effect of GloMPO’s supervision and control machinery. These tests aim to demonstrate that:

1. Forced termination of optimizers results in a more efficient use of an evaluation budget; and,
2. Information sharing between optimizers through the manager increases performance.

#### Optimization task

Test A explores various global optimization test functions. Such functions are typically very quick to evaluate, and thus allow us to test a wide array of configurations to demonstrate that the management effect is robust. They have, however, been rightly criticized in the past for not providing a sufficient challenge for state-of-the-art optimizers [4, 8, 39, 51] and creating the incorrect impression that difficulty scales with dimensionality.

In the scope of this test, however, we are not interested in identifying a GloMPO configuration that competes with state-of-the-art optimizers and consistently finds the global minimum. The aim here is to demonstrate that an optimizer which struggles on a particular function, can benefit from GloMPO’s management aspects of information sharing and early termination. As will be clear from the results, the CMA-ES optimizer used here did struggle with these functions and did not find the global minimum with any regularity. As a first step in our work, we did not judge adjustments to the traditional functions to be necessary.

Four test functions are used in this work. Brief descriptions are provided below, and the interested reader can consult the Supplementary Information for visualizations and the explicit functions (Additional file 1: Section S1):

- *Rastrigin* The Rastrigin function [29] is globally unimodal around the minimum, but the surface is highly oscillatory. Population-based optimizers can be expected to rapidly near the origin (where the global minimum is located), but then get trapped in the nearby local minima when their search radii begin to shrink. Tested in 66 dimensions.
- *Deceptive* The Type III Deceptive test function [52] is particularly challenging because there is a very small basin of attraction around the global minimum. The region immediately surrounding it is sloped away from the global minimum to various local minima. The location of the global minimum, which is customizable in the function, was placed randomly each time it is used in this work. Tested in 20 dimensions.
- *Schwefel *The Schwefel function [33] has several features making it particularly difficult to optimize: (1) unlike the Rastrigin test function, it does not have a global gradient leading optimizers to the minimum; (2) it has a much larger search domain; (3) the global minimum is hidden near the boundaries where the function becomes more oscillatory; and (4) the second best solution is located very far away from the best. Tested in 20 dimensions.
- *Shubert* The Shubert function is highly multimodal with degenerate and periodically distributed global minima (i.e., equal function values at different locations in parameter space). Very good second-best solutions immediately surround each global minimum entrapping many optimizers. It is investigated here to test the ability of different optimization strategies to identify multiple degenerate global minima. Tested in 4 dimensions.

#### Child optimizers

CMA-ES is selected as the child algorithm in these tests. This is a popular global optimization strategy, suited to a wide array of problems. A particularly appealing property of this algorithm is that it has only a few hyper-parameters, with sensible defaults for most as functions of the dimensionality of the problem [25]. A further consideration was the fact that this optimizer proved most efficient in locating good minima in the reparameterization of ReaxFF force fields in the work of Shchygol et al. [58]; this optimization challenge appears in “Test C: GloMPO on ReaxFF”.

The implementation used is adapted from the Python package available at Hansen et al. [27]. Most settings are unchanged from the defaults set in this package. The initial setting for the parameter governing how far the algorithm can explore from the incumbent solution ($\sigma _0$) is set to half the distance between the upper and lower bound (Eq. 1)—which is the same in all dimensions for the test functions. $\sigma _0$ is purposely broad to make the initial starting location uninformative, and force a very global search.

1 $$\begin{aligned} \sigma _0 = \frac{x_{max} - x_{min}}{2} \end{aligned}$$

To investigate GloMPO’s ability to share information between its children, CMA-ES is used in a second way to make it compatible with receiving outside input. It has been previously shown that injecting good solutions into CMA-ES’s population can be very effective at improving its performance [24]. We extend this further using the GloMPO framework to dynamically share good iterations between CMA-ES instances.

Algorithm 2 shows the architecture of this approach. A parameter vector producing a very low function value is seeded to the algorithm, and every several iterations this candidate is forced into the next iteration’s sampled population. Practically, an incumbent solution is seeded by selecting the best ever solution from previous optimizers as the starting location. If an improved solution is found, the injected candidate is updated. The result is that the algorithm can still maintain a wide search radius but is unable to move its mean too far away from the good solution. This injection is a form of elitism, but, in practice, does not result in the same loss of exploration that true elitism does. The injections act to nudge the algorithm’s mean back towards the good solution, hence the name given hereafter: nudging-CMA or N-CMA. In a managed setting, the updates to the injected candidate may also be obtained from external sources i.e., other children GloMPO is managing. 
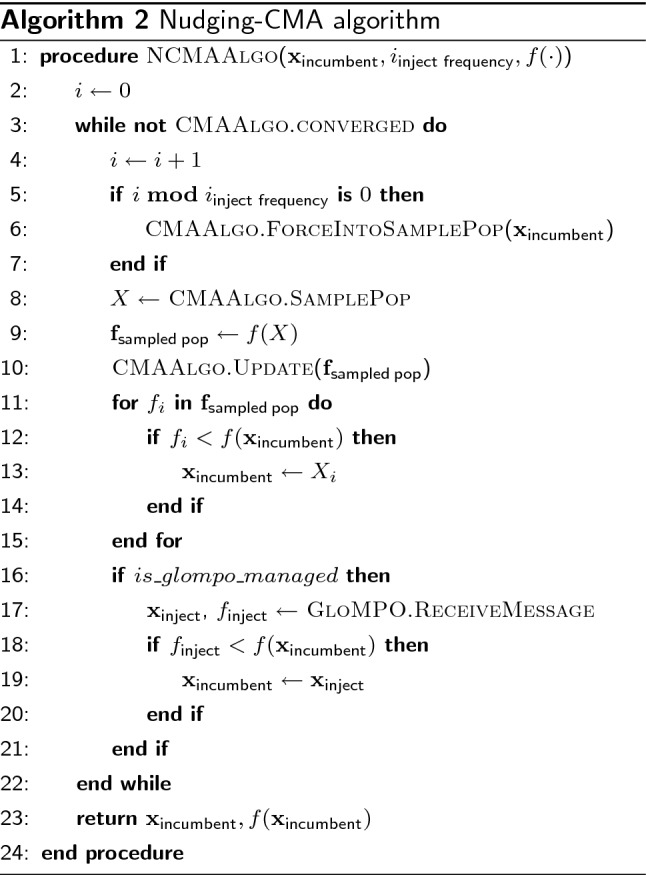


#### Other settings

Four other settings are also investigated. They include the number of optimizers used by serial and GloMPO optimizations, and their convergence settings, which in this case refers to the function tolerance. These are primarily explored to illustrate GloMPO’s robustness to such changes.

Two generators are used. This refers to the starting guesses for the optimizers. Generally, this was selected by uniformly sampling from the parameter space. In most of the configurations using N-CMA, the optimizers were started at the manager’s incumbent solution, and this also forms the initial nudging candidate. The alternatives for all these settings are given in Table 2.

**Table 2:** Summary of other configuration settings

Property	Tested values	Comments
Convergence	10$^\text {-6}$, 10$^\text {-11}$ and 10$^\text {-20}$	Refers to the tolfun convergence setting of the individual CMA optimizer instances
Max Serial Jobs ($n_s$)	5, 10, 15 and 20	Number of unmanaged optimizers run in a single bout
Max GloMPO Jobs ($n_g$)	2, 4, 7 and 10	Number of managed optimizers alive at any moment during a GloMPO managed bout. Note, this is not the total number of optimizers used as GloMPO may replace any of its children at any time
Generator	Random, Incumbent	Random: uniformly randomly selected point in parameter space. Incumbent: best point seen thus far by the manager

#### Benchmark test configurations

Testing every combination of the above settings would not be practical. The configurations actually selected for testing are built up methodically. We believe them to be generally representative, but, of course, we are only able to test a small fraction of all possible configurations, which are themselves a random subset of an infinite set of possible configurations. In total, 48 combinations are tested in 4800 bouts. Each set of bouts is listed in Additional file 1: Table S1 of the Supplementary Information and given a set identification number.

The first configuration tested is Set 12 which uses the Schwefel function (objectively the hardest function as outlined in “Optimization task”), default CMA-ES settings, random start locations, and ten and four serial and GloMPO optimizers respectively; the latter two settings chosen *ad hoc*. From this configuration, tests are performed by changing the number of serial optimizers, and then the number of GloMPO optimizers are changed. Returning to Set 12, the convergence settings are changed. The other test functions are then tested at the highest and lowest convergence settings. The N-CMA tests follow the same pattern of changing only one setting at a time, but only the Schwefel function is studied.

It should be emphasized that the choices we made for Test A with regards to the generator, large initial optimizer search radius etc. are purposely not very sophisticated ones. The aim here is to isolate the effect of hunting as far as possible, so that differences in performance can be solely attributed to that effect. We did not attempt to tune GloMPO configurations to become competitive with state-of-the-art optimizers on these functions.

### Test B: GloMPO as a framework

The aim of Test B is to demonstrate a more sophisticated GloMPO configuration. It shows that GloMPO is flexible enough to mimic popular and efficient metaheuristic algorithms and, combined with the advantages of management demonstrated in Test A, produces better results than the unmanaged counterpart.

#### Child optimizers

For this test we make use of two very popular, and effective metaheuristic algorithms; dual annealing (DA) [69] and basin-hopping (BH) [67]. We have selected these partially because they have been implemented in Python’s SciPy library [66]. This package is extremely popular, and a first port of call for non-experts looking for certain mathematical routines (like optimizations).

These routines may no longer be state-of-the-art, but they remain popular because their algorithms are quite intuitive. Also, in the context of this test, we are most interested in demonstrating how such metaheuristic algorithms can work within the GloMPO framework.

Algorithm details for the two routines used here are included in Additional file 1: Section S3. Broadly speaking, both algorithms use a Monte Carlo step-taking algorithm as their metaheuristic and launch periodic local search algorithms. BH launches local searches every iteration, while DA does so more infrequently based on internal decision criteria. Unless otherwise stated in Table 3, the default settings of the SciPy v1.2.1 implementation are used.

**Table 3:** Customized settings used for the basin-hopping and dual annealing algorithms

SciPy parameter name	Description	Value	Comment
Basin-Hopping			
T	Temperature	0.8	Changed to match the values used by Wales and Doye [67]
stepsize	Maximum step in each dimension that can be taken by the random displacement	1	
niter	Number of Monte Carlo steps and local optimizations	100	5000 was used by Wales and Doye [67] but their results show that global minima were often found in the first few hundred iterations. Since we are not interested in actually obtaining the global minimum, we select a value of 100 to make the cost of the optimizations bearable. This is sufficiently long in lower dimensions, to locate the global minimum, and sufficiently long in higher dimensions to make a fair comparison of performance
Dual Annealing			
initial_temp	Initial temperature	50000	Governs the maximum step the random displacement can take. Increased from the default to make the optimizer more exploratory since early test work showed a propensity to get stuck in the first minimum located
restart_temp_ratio	Ratio between current and initial temperatures which resets the temperature to the initial value	0.01	Increased from default to actually trigger new restarts and force the optimizer to explore other minima

#### Test strategy

The “Benchmark test procedure“ is configured for Test B as follows:

1. The ‘serial’ run used a single execution of the BH or DA routines as implemented in SciPy. A single repeat ($n_s = 1$) was used since the aim of these experiments was to investigate a metaheuristic strategy’s performance with and without GloMPO management and information sharing. To verify that GloMPO’s performance cannot be attributed solely to multiple start locations, some tests are repeated with $n_s=4$.
2. The GloMPO run splits the metaheuristic into their ‘upper’ and ‘lower’ routines. The upper algorithm is a Monte Carlo-based step procedure, and the lower one is a BFGS local optimization. The upper routine is used as a ‘generator’ to identify starting locations for child optimizers (the lower routine).
3. As before, both competitors are limited to the same number of function evaluations.
4. The GloMPO generators are designed to match their parent algorithm as closely as possible, but some modifications were required to support GloMPO’s asynchronous parallel behavior since both upper-level algorithms are sequential. Details have been provided in Additional file 1: Section S2.
5. Tests were repeated in 30, 75, 150 and 225 dimensions. Full configuration details are given in Additional file 1: Table S2.

The distinction between serial and GloMPO configurations is that the GloMPO configuration runs local optimizations in parallel, has the power to terminate them early, and centralizes information from multiple sources into a single generator step.

#### Optimization task

In this test, we choose to make use of a more challenging real-life global optimization challenge: the optimal arrangement of particles in a Lennard-Jones (LJ) energy potential [67]. This has the advantages of being harder than the previous test functions, but still cheap enough to be optimized many times.

Whenever two atoms approach one another in space, they experience an attractive force pulling them together. As the distance between them decreases, so does the force of attraction. At a critical distance, the atoms begin feeling a repulsive force which typically increases very steeply. The simplest way to describe this interaction is through the use of the Lennard-Jones energy potential in Eq. 2 where *E* is the potential energy of the particle arrangement, *X* is the matrix of *d*-dimensional Cartesian coordinates describing the location of *N* particles, $\epsilon$ is the depth of the energy minimum, $\sigma$ governs the location of the minimum, and $r_{ij}$ is the Euclidean distance between atoms *i* and *j*.

2 $$\begin{aligned} E(X) = 4\epsilon \sum _{i<j}\left[ \left( \frac{\sigma }{r_{ij}}\right) ^{12} - \left( \frac{\sigma }{r_{ij}}\right) ^6\right] \end{aligned}$$

The optimization problem is to find the arrangement of particles which minimizes the energy in Eq. 2. The dimensionality of the optimization problem is *Nd*, thus, the 30-, 75-, 150- and 225-dimension problems optimized here had 10, 25, 50 and 75 atoms, respectively.

The Lennard-Jones potential energy surface is characterized by many minima located near steep and non-finite regions. Due to the fact that a translation, rotation or permutation of particles will not change the energy value, the surface has very many degenerate global and local minima. In our tests, as is commonly done in literature, we have set the parameters $\epsilon = \sigma = 1$.

The BFGS local optimization strategy was given access to analytical derivatives; thus, this is not an HEB problem since it is not black-box. We make this choice to give the serial optimizers the best performance possible. If the derivative function were not made available to the local optimizer, a numerical approximation would be constructed by finite differences. This increases the evaluation cost and produces longer optimizer tails which GloMPO could potentially terminate. In our studies, not using analytical gradients improved GloMPO’s performance in comparison to the serial optimizer even further than the results included here.

### Test C: GloMPO on ReaxFF

Test C is a demonstration of GloMPO on a real-life HEB optimization problem; the reparameterization of a ReaxFF force field.

#### Optimization task

Within the study of computational chemistry, many approaches exist to calculate the energy of a chemical system. These approaches can be broadly divided into three categories. The first group of methods, known as *ab initio* methods, comprises models which are fully based in theory and can be solved from atomic positions and physical constants alone. *Ab initio* methods are generally the most accurate but involve extremely complex calculations. Methods which introduce some empirical approximations, but still follow the Hartree-Fock formalism, are known as semi-empirical methods. Those that totally abandon this formalism are known as empirical models.

Empirical and semi-empirical models serve an invaluable role in allowing computational chemists to model temporal and spatial scales unobtainable with *ab initio* approaches. By their very nature, these methods introduce empirical parameters into the calculation of the potential energy surface (PES). The use of such parameterized models greatly decreases the cost of the calculation but creates a problem of identifying the appropriate parameters to be used.

ReaxFF is an example of an empirical method (sometimes also called a force field) which represents the state-of-the-art approach to simulating chemical reactions at scale. ReaxFF was first introduced for hydrocarbons in Van Duin et al. [65] and, since then, has been successfully extended to many different chemical systems [3, 31, 38, 40, 56]. To model a phenomenon as complex as reaction, ReaxFF introduces global parameters, parameters for chemical elements and pairs, triplets, and quadruplets of elements, many of which have no physical interpretability. The total number needed can quickly become unwieldy with tens or hundreds being required by some models. There is often very little insight as to which values or range of values are appropriate. Optimizing ReaxFF force fields for different chemical systems is a significant hurdle to its wider application; one which is getting more attention in recent years [17, 22, 30]. ReaxFF is used in this work as an archetypal example of a pernicious fitting problem.

To find appropriate values for all the ReaxFF parameters, the computational chemist must create a training set (${\varvec{y}} \in {\mathbb {R}}^n$ where *n* is the number of items in the training set) containing energies, forces, bond angles, bond distances, or any other property they identify as important for the field to replicate. These values are obtained from several clusters of atoms calculated using higher level methods, or from experimental results. Each item of the training set has a corresponding set of input conditions, such as atomic positions ($X \in {\mathbb {R}}^{m \times n} = [{\varvec{x}}_1, {\varvec{x}}_2, \dots , {\varvec{x}}_n]$).

Construction of the training set is itself a non-trivial problem. The computational chemist must ensure that: (1) the set sufficiently samples all the areas of interest of the energy landscape without introducing overly sensitive items; (2) important items are correctly weighted; and (3) a low evaluation cost is maintained.

Corresponding values to those in the training set are estimated by the model ($f({\varvec{x}}; {\varvec{p}}) :={\hat{y}}$) by selecting values for each parameter (${\varvec{p}} \in {\mathbb {R}}^l$). The deviations between the training set values and those estimated are then used to generate a cost function ($E({\varvec{p}})$). The type of cost function shown in Eq. 3 is the sum of square differences as it is the most common choice, but other constructions such as sum of absolute differences have also been used. The $\sigma _i$ values in the cost function represent a scaling factor to make contributions of different units comparable. Increased or decreased importance can be attributed to certain items through the use of individual weights ($w_i$).

3 $$\begin{aligned} E({\varvec{p}}) = \sum _i^n \left[ \frac{w_i(y_i - {\hat{y}}_i)}{\sigma _i}\right] ^2 = \sum _i^n \left[ \frac{w_i(y_i - f({\varvec{x}}_i, {\varvec{p}}))}{\sigma _i}\right] ^2 \end{aligned}$$

Finding the best parameters becomes a task of minimizing the cost function. Despite the importance of this optimization step, it remained, until recently, a poorly addressed problem. For several years, the default approach was the sequential one-parameter parabolic extrapolation (SOPPE) method [38, 64] (also called SOPPI [17]) which, as the name suggests, tunes parameters individually while fixing the other terms. However, this method does not adequately account for correlation between terms, and many iterations are needed to find a suitable parameter set [17, 38]. The method itself is also impenetrable to non-experts as the order in which parameters are optimized is critical to obtaining a satisfactory final set of parameters. Other publications rely only on the author’s expertise and adjust the field manually [2].

More recently, however, workers have attempted to tackle the problem systematically and introduce more robust optimization algorithms. Larsson et al. [38] applied genetic algorithms with some success to parameterize a SiOH force field. Furman et al. [17] introduced a particle swarm-based technique called RiPSOGM. Trnka et al. [62] applied the covariance matrix adaptation evolutionary strategy (CMA-ES) to generate force fields for enzymatic reactions. Hubin et al. [31] and Iype et al. [32] applied Monte Carlo simulated annealing methods to optimize their force fields. Hu et al. [30] and Stepanova et al. [60] also introduced novel techniques using unique cost functions, and Guo et al. [22] has developed a machine learning-based parameterization technique. Finally, Shchygol et al. [58] conducted a review of several of the aforementioned approaches and determined that CMA-ES is generally the best performing, but it could still not be relied upon to perform consistently when repeated on the same problems several times.

The two force fields selected for reparameterization in this work are taken from Shchygol et al. [58] and configured in the same way.

- *Cobalt* This is a force field describing liquid and solid cobalt, first developed in Labrosse et al. [37]. Twelve parameters in the model are configurable. The field is reparameterized against 144 training points which are all reaction energies.
- *Disulfide* The second force field is taken from Müller and Hartke [43] and describes disulfide structures. The reparameterization attempts to optimize 87 parameters against 4875 training points, which are a combination of atomic charges, geometries, cell parameters and reaction energies. Given the greater complexity of the training set, and larger number of parameters, this force field represents a much greater challenge than the cobalt one.

The principal difficulty during ReaxFF reparameterization efforts is that the cost function is a black-box global optimization problem. Although an explicit functional form exists, its evaluation usually contains non-robust steps [58]. In addition, although evaluating its analytical derivative may be theoretically possible, in practice existing ReaxFF implementations do not support them. Most implementations primarily focus on computational efficiency for molecular dynamics simulations instead [56]. Only recently, derivatives towards ReaxFF parameters were realized by a re-implementation of ReaxFF from scratch, making use of automatic differentiation in TensorFlow. This proof-of-concept was limited to training data consisting only of single-point energies [22]. Other frameworks have attempted to redesign the formalism to ensure smoother energy surfaces [19]. In general, however, the ReaxFF cost function is a rugged function with many discontinuities [11], such that derivatives can be ill-defined or are of limited use for parameter optimization.

#### Benchmark test configuration

For the most part the benchmark test was configured as done in Test A. Random initial guesses were used with CMA-ES child optimizers configured with a wide initial search radius. However, given the expense of these optimizations, only ten bouts were repeated per configuration, and only three configurations were tested on each force field: (1) strict hunting only, (2) looser hunting only, and (3) hunting and information sharing using N-CMA. Other settings were selected based on the best results from Test A.

‘Loose’ and ‘strict’ hunting configurations refer to how aggressively GloMPO shut down child optimizers. A ‘Loose’ hunting style allowed optimizer to remain alive for longer, well into the focus phase. ‘Strict’ hunting terminated the optimizers as soon as they began to appear to focus. More details about the exact bout configurations can be found in the optimization results files.

We have chosen to keep the configuration simple and straight-forward for this demonstration. However, we have plans to publish more sophisticated GloMPO -managed search strategies in a subsequent paper dedicated to ReaxFF reparameterization.

### Software

GloMPO v2.0.5 was used for the sets using only CMA-ES optimizers, and v2.1.0 was used for those using nudging-CMA in Tests A and C. GloMPO v 3.1.1 was used for Test B. The code is available open source under the GPL-3.0 license [16].

GloMPO comes bundled with an interface to the new ParAMS [35] tool in the official release of AMS2020.1. ParAMS, in turn, interfaces to the ReaxAMS [6, 55, 65] engine. In ReaxAMS, geometry optimizations are done with the FIRE optimizer as opposed to the L-BFGS algorithm used by classic ReaxFF. All settings in the ReaxFF control files are converted to equivalents in ReaxAMS and FIRE using built-in ParAMS converters.

## Results and discussion

### Test A: advantages of management

As mentioned previously, a total of 4800 bouts were performed for Test A. We define a bout victory as GloMPO finding a lower function value than its serial counterpart. The win percentage is the fraction of bouts GloMPO won over the 100 bouts in a set.

In the presentation of these results, the success percentage of each set is calculated, and these results are pivoted along the various axes of interest such as convergence, task, number of optimizers etc.

#### Hunting only

**Fig. 4:**
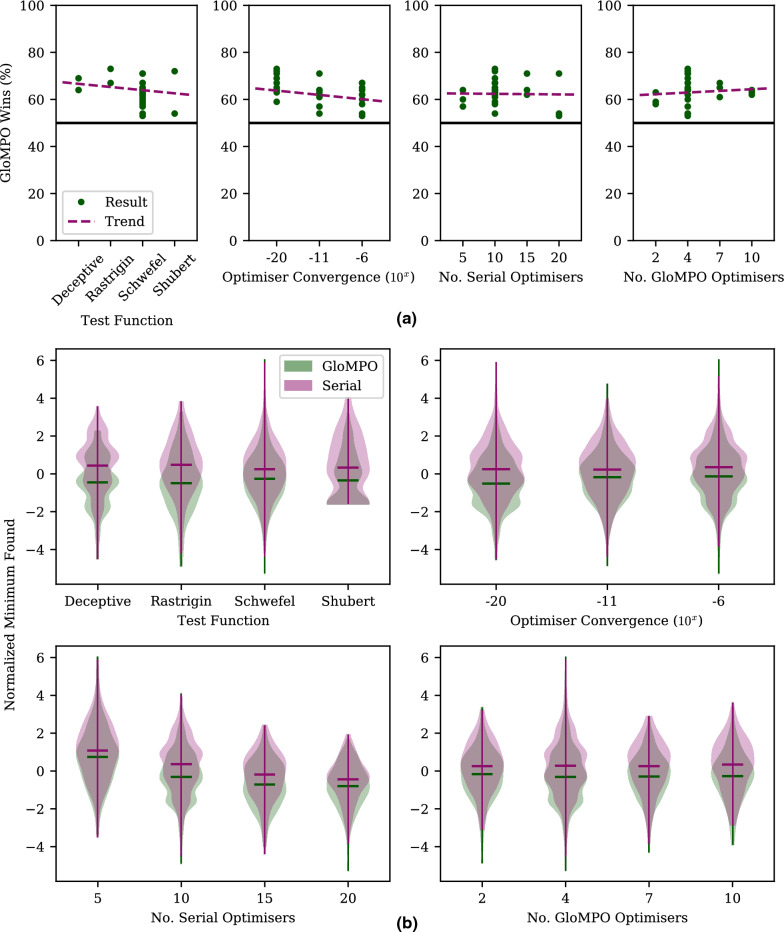
**a** GloMPO Test A win rates grouped by configuration setting. Trendlines shown in purple, 50% rate marked by black solid line. **b** Final minima found by serial and GloMPO optimizations grouped by different configuration settings and shown in violin plots. Minima have been normalized by function average and standard deviations to make them directly comparable. Mean values shown by corresponding colored solid bars. Sets involving N-CMA are excluded for separate discussion

Figure 4a shows the win rates grouped by different configuration settings. To be clear, the data is the same in each plot, just grouped in different ways. Sets involving N-CMA have been excluded from this figure for later discussion so that the effect of supervision and termination can be studied in isolation. Overall, averaging across the remaining 27 configurations, GloMPO won on average 62 ± 6% of the time and drew 3% of the bouts, demonstrating a modest benefit generated by the managed optimization approach.

Figure 4b shows violin plots of the final minima found by serial and GloMPO optimizations for each of the 2700 bouts in the truncated group of sets as described above. Again, the data is grouped into different configurations. Given that each function explores different values, the minima have been normalized by function type to make them comparable.

The overall success rate masks several important configurational effects. The number of serial optimizers has the effect of increasing the overall number of evaluations used, but also provides more opportunities for serial optimization to identify different minima. Using an increasing number of serial optimizers shifted the final minima down for both optimization approaches due to the higher iteration budget. However, in all cases GloMPO’s win rate remained mostly unchanged, and it was always able to produce distributions with lower values than serial optimization, an effect more pronounced with a higher number of optimizers. In other words, repeating an optimization over and over again in a serial manner increases the chances of finding a better minimum, but GloMPO is more likely to find an even better one in the same amount of time. Of course, there are caveats to this. At the lower limit of one or two serial optimizations, GloMPO would perform poorly as it distributes its very limited budget between several optimizers without enough time for any of its children to sufficiently develop. At the upper limit, where very many optimizations are repeated, the serial approach is bound to yield better or the same minimum as GloMPO, simply by statistical probability. However, the calculation time required in this scenario makes it unrealistic.

In terms of the number of GloMPO optimizers alive at any one time, there are slight decreases in win rate at the lowest and highest values tested. When $n_g = 2$ there are too few points of comparison for GloMPO to dynamically reject an optimizer and start another one, i.e., the optimization is nearly serial. At the other limit, $n_g = 10$, the iteration budget is used too quickly. However, this effect is a very small one, and barely noticeable in Fig. 4b. This suggests that GloMPO may be quite robust to configurational changes. The user can, for example, configure GloMPO based on computational resources without being overly concerned about the impact on results.

The greatest performance impact comes from the optimizer convergence setting (Fig. 4a, second panel). In these sets, GloMPO generates its performance improvement by limiting wasted time in bad minima. At lower convergence tolerances, optimizers naturally spend less time in any minima, thus limiting GloMPO’s opportunity. The effect, however, is not very pronounced across the range of fourteen orders of magnitude tested. While $10^{-20}$ may seem like an excessively tight tolerance, the mean value produced by serial optimizers with this tolerance is 38% lower than that produced by serial optimizers with a tolerance of $10^{-6}$. This big difference cannot be explained by greater numerical precision alone. Although higher tolerances do force optimizers to search for longer, the result is not limited to mining more decimals places. It also provides more opportunities for optimizers to identify other and better minima. This is not generally true, but true for the population-based optimizer used here.

Finally, the distributions and win rates are considered as a function of optimization task. As mentioned previously, the functions tested all exhibit different forms of multimodal behavior. In all cases GloMPO performed better than serial optimization. In the case of the Shubert function, tested in only four dimensions, the low dimensionality and periodic degeneracy makes finding the global minimum relatively easy. However, GloMPO was still able to produce a better distribution of results.

#### Information sharing

Optimizer control is the most basic type of management of which GloMPO is capable, and it has been demonstrated to be effective in producing better optimization results. Further improvements are possible when GloMPO shares information between its children. The manager informs its children of the best point ever seen whenever this is updated. Optimizers may then use this information in any way they wish. Coupling this GloMPO ability with Nudging-CMA (see “Child optimizers”) is particularly powerful as it results in the group of managed optimizers working collaboratively by sharing their results and using them as new nudging vectors in real-time.

**Fig. 5:**
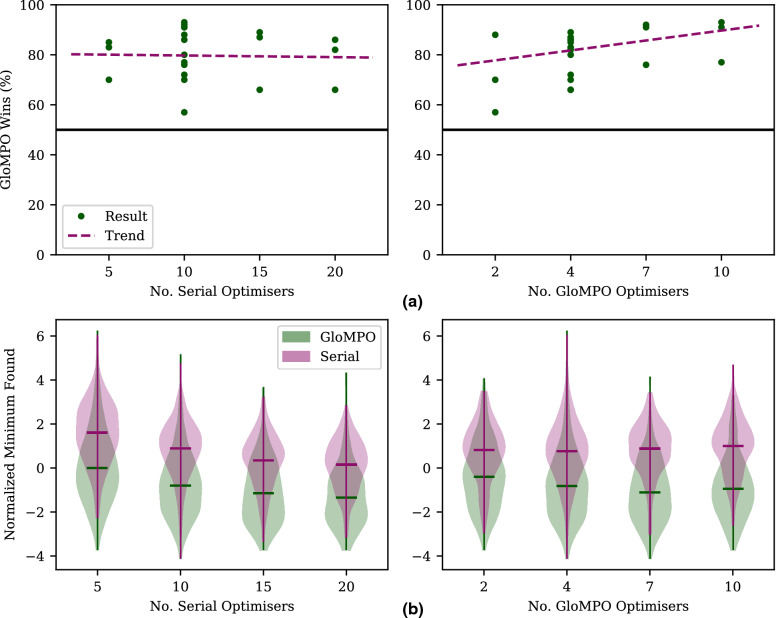
GloMPO win percentages and minima distributions for Test A sets using N-CMA. **a** GloMPO win rates grouped by different configuration settings. Trendlines shown in purple, 50% rate marked by black solid line. **b** Final minima found by serial and GloMPO optimizations grouped by different configuration settings and shown in violin plots. Minima have been normalized by function average and standard deviations to make them directly comparable. Mean values shown by corresponding colored solid bars

Figure 5 shows the distribution and win percentages of the 2100 bouts performed with nudging. The improvement in performance is dramatic. GloMPO is able to win 80 ± 10% of the bouts (0.4% draws), 17% more than the sets using CMA-ES. The margin of the wins is also much larger, as evidenced by the great distance in distributions between serial and GloMPO optimizations.

One feature of particular interest is the strong effect of the number of GloMPO optimizers. Previously, using too many or too few optimizers at once in a managed optimization had a small detrimental effect on performance. With information sharing, however, using more optimizers at once increased the amount of collaboration between them, and increased the GloMPO success rate to 92% (no draws). The effect of the number of serial optimizers, however, remains unchanged reinforcing the robustness of GloMPO.

Included in these results are sets in which serial optimization is configured to run with N-CMA. This is an impractical way to optimize in general but is tested here to isolate the effect of GloMPO’s management as far as possible. In this setup, optimizers are started sequentially rather than simultaneously. Each subsequent optimizer is started at the best point seen thus far and nudged according to the N-CMA algorithm (Algorithm 2) during its run. In this configuration GloMPO still achieves a win rate of 69 ± 7% (0.4% draws), similar to the win rate when using normal CMA-ES. This is evidence that N-CMA alone is not responsible for the performance improvement. Rather, the information sharing and collaboration provided by the GloMPO system plays an important role. As mentioned, this is not a practical optimization strategy, and if normal serial optimization is compared to GloMPO using N-CMA the win rate increased to 86 ± 6% (no draws).

The frequency with which the injection is done is absolutely essential to the success of N-CMA itself. Note, the important distinction between the frequency with which GloMPO shares information between its children (which occurs whenever a new best solution is found), and the frequency with which the CMA algorithm injects that parameter set into its sampling. Here, we refer to the latter. When the injection is done too frequently, the optimizer is forced to converge to the injected point. Conversely, when too infrequent, the algorithm often becomes stuck in an endless exploratory loop and never converges. The range of frequencies for which the technique works also seems very narrow; our testing showed injections every 10 iterations to be effective.

Nudging-CMA is not suited to trap functions like the Deceptive function presented in “Optimization task”. Consider a simpler example with the same behavior: a function *f* with the following minima:

4 $$\begin{aligned} f(1, 1, 1, 1)&= 0 \end{aligned}$$

5 $$\begin{aligned} f(0, 1, 1, 1)&= 4 \end{aligned}$$

6 $$\begin{aligned} f(0, 0, 1, 1)&= 3 \end{aligned}$$

7 $$\begin{aligned} f(0, 0, 0, 1)&= 2 \end{aligned}$$

8 $$\begin{aligned} f(0, 0, 0, 0)&= 1 \end{aligned}$$

An increasing number of zeros in the parameter vector decreases the function value but the true global minimum is located at $x=\{1, 1, 1, 1\}$. It is impossible to know *a priori* if a function exhibits this type of behavior. However, in high-dimensional problems, this nudging behavior can nevertheless help find better local minima, even if it does prevent one from finding the global minimum.

For example, in optimizations of the 20D Schwefel function using normal CMA-ES, optimizers often converged to points in which 9 to 15 elements equal 420.9687; this is near the global minimum in which all elements equal 420.9687. Using these vectors as nudging candidates helps guide optimizers to better points in which 17 to 20 elements are correctly identified. In this case the more parameters correctly set, the better the function value. For N-CMA to work in this way the function must produce a lower value for every parameter which is correctly set. This, in turn, relies on a weak statistical correlation between parameters.

### Test B: GloMPO as a framework

**Fig. 6:**
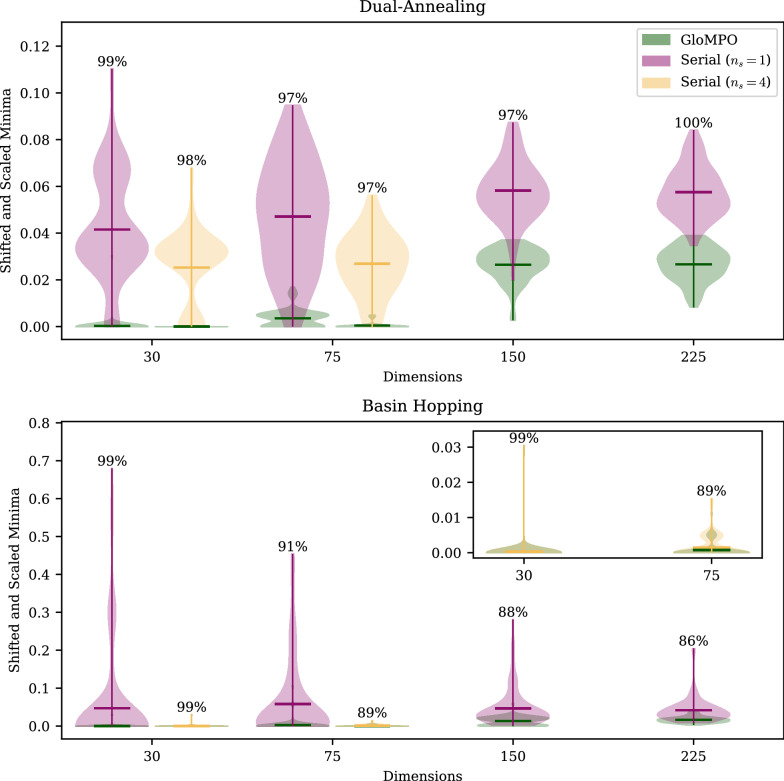
Distributions of minima located through serial and GloMPO using dual-annealing and basin-hopping strategies on the Lennard-Jones problem of varying dimensions. GloMPO win rates included as annotations. Function values shifted to make them comparable such that ${\tilde{f}} = \frac{f - f_\text {global min}}{|f_\text {global min}|}$

The aim for Test B was to demonstrate through a simple example that GloMPO could mimic and outperform some popular metaheuristics through its framework. The BH and DA algorithms selected for this were applied to the LJ optimization problem. The distributions for the 100 bouts of each configuration are shown in Fig. 6 along with the GloMPO success rate. To make the results comparable over the multiple dimensions in which they were run, function values have been shifted and scaled according to Eq. 9 such that zero is the known global minimum.

9 $$\begin{aligned} {\tilde{f}} = \frac{f - f_\text {global min}}{|f_\text {global min}|} \end{aligned}$$

For $d=30$ (10 atoms) and $d=75$ (25 atoms), the tests were repeated with $n_s=1$ and $n_s=4$, i.e., the number of serial BH or DA optimizers run. The head-to-head ($n_s=1$) tests directly compare the metaheuristics, but the GloMPO competitors had the advantages of information sharing and supervision demonstrated in Test A; in these tests GloMPO competitors were run with four parallel children ($n_g=4$). The $n_s=4$ tests were performed to demonstrate that GloMPO’s performance could not be solely attributed to the fact that, in the head-to-head tests, GloMPO effectively had four random start locations while the serial competitor had one.

Figure 6 shows very strong performance by the GloMPO configurations with win rates remaining remarkably high for both metaheuristics across most tests. For the most part, GloMPO distributions regularly included the global minima and had significantly fewer outliers than the serial configurations. We believe that the improved performance compared to Test A can be attributed to the more sophisticated generators used here.

Analyzing $n_s=4$ for the DA runs, one can see that the extra optimizers had the expected benefit of reducing the mean result and narrowing the distributions somewhat, but the serial results were still poor. For the BH runs, the serial results were dramatically improved, and the distributions were almost the same as the GloMPO ones. The effect of increasing the number of serial BH optimizers is (almost) the same as running a single optimizer for a longer period. In that respect the results are unsurprising. As mentioned in Table 3, the serial optimizers were limited to 100 local searches, somewhat short compared to some literature values.

**Fig. 7:**
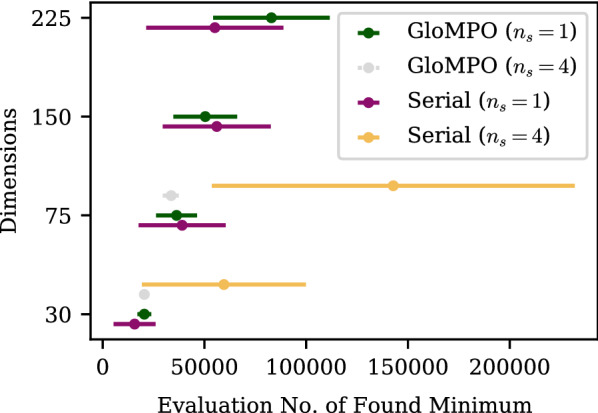
Mean and standard deviation of the evaluation number of the minimum found through serial and GloMPO using the basin-hopping strategy on the Lennard-Jones problem of varying dimensions

One may be tempted, in that case, to dismiss GloMPO’s performance as unimportant since it can be replicated by simply running serial optimizers for longer. Consideration should, however, be given to the efficiency with which the evaluation budget is used. We reiterate that serial and GloMPO share the same limit on function evaluations, but the final evaluation need not be the best value ever seen, i.e., the lowest minima can be found at any point during the optimization. Figure 7 shows the average and standard deviation of the point in time (the function evaluation number) at which the minimum was located for the BH tests across the various configurations and averaged over each of the 100 bouts.

For the $n_s=1$ tests, the number of function evaluations needed to find the minimum were comparable between GloMPO and serial as expected. Serial occasionally found its best minimum sooner because of the inherent GloMPO cost of running parallel children; but we recall the minima it found were much worse. For the $n_s = 4$ tests, serial was able to find comparably good minima but far later than GloMPO. At higher dimensions it is unlikely that $n_s = 4$ will be sufficient to remain competitive with GloMPO and this would need to be increased further, thus increasing the expense of the optimization.

In the case of fast functions, one may be indifferent to this extra expense, and wish to eschew the GloMPO overhead for the simplicity of simply running multiple serial optimizations. It is for this reason that we are particularly focused on HEB functions, where the difference in wall time becomes significant.

It is also worth mentioning that, during the BH $n_s=1$ tests, we noticed that the GloMPO configuration was starting an extremely large number of local optimizations at a single minimum towards the end of the optimization because it had only been configured to stop when it had used the same number of function evaluations as the serial optimizer. To avoid repeating this problem (and unnecessarily bloating results files) the GloMPO competitors for the $n_s=4$ tests were given an extra stop criterion: the total number of local searches was limited to the same number conducted by the serial tests, i.e., 400. Even with this extra limitation, GloMPO was able to outperform the serial competitors.

As a final word, we emphasize that the point of these tests is not to identify a new best optimizer for LJ clusters. This has been the topic of much literature and focused attention by others [9, 49, 50, 67]. We seek to demonstrate that, in general, GloMPO is very customizable, and can improve the performance of optimizers on a problem, particularly in the context of novel HEB problems, where the optimal optimization approach is not clear.

### Test C: GloMPO on ReaxFF

The final test we present in this work demonstrates the utility of GloMPO on a real-life HEB; the reparameterization of a ReaxFF force field. We aim to show here some of the qualitative advantages of the framework.

#### Timings

The first analysis we conduct is the overhead cost of GloMPO. Its design intent is for expensive functions, and having a measure of when it becomes a bottleneck is useful to deciding if a function is appropriate for GloMPO management or not. We begin by analyzing the behavior of the ReaxFF cost functions, before timing them within GloMPO.

**Fig. 8:**
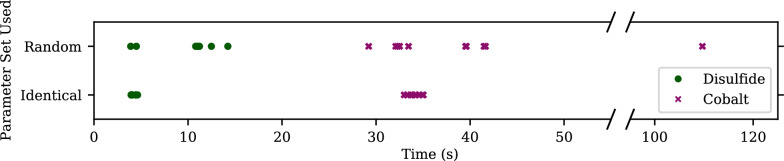
Evaluation times of the ReaxFF cost functions using random parameter sets and repeatedly evaluating the same set (ten times each). Evaluations were performed on a single 1.30GHz Intel Core i7-1065G7 CPU

The evaluation of the ReaxFF cost function is relatively expensive and quite variable. For context, Fig. 8 shows timings of a single cost function evaluation for the cobalt and disulfide cost functions evaluated on a single 1.30GHz Intel Core i7-1065G7 CPU. In each scenario, ten repeats were performed. Two setups are shown. First, to demonstrate the repeatability of the timing for a given parameter set, the cost functions were evaluated at the midpoint of all the bounds. Second, to demonstrate that the evaluation time is a function of the parameter set, the cost functions were evaluated ten times each with randomly generated parameter sets.

Both cost functions perform reproducibly and show little variation when repeatedly evaluating the same parameter set, however, they show large variability when evaluating different ones. The difference can be attributed to certain geometry optimization taking longer to converge when the parameter set is a poor one. This can become a serious problem when using a population-based optimizer like CMA. The optimizer can only proceed as fast as the slowest evaluation, in cases where the timings differ substantially, this results in a significant amount of idle time within the optimizer (if the function evaluations are evaluated in parallel). Notably, the cobalt function is much more expensive and variable than the disulfide one, despite being conceptually simpler. This is an unfortunate consequence of how the training set is evaluated within ReaxAMS.

Table 4 quantifies the overhead of using GloMPO in conjunction with the disulfide and Schwefel functions. Cobalt was excluded from this test because its variability in evaluation time would make it difficult to isolate GloMPO’s effects. In each scenario GloMPO managed a single optimizer and let it run for 300 s, the total number of function evaluations were counted. Each scenario was repeated five times, and the average and standard deviation is shown. In such cases a single optimizer was run using a threaded backend. Python’s global interpreter lock implies that the entire process is run through a single core. For comparison, the same optimizer, given the same task and time limit, was run outside of GloMPO. The ‘optimizer’ in these tests was not a real optimizer but rather an infinite loop that continuously evaluated the same vector. This eliminated optimizer related overhead, some ReaxFF variability, and ensured there would be no convergence before the end of the time-limit. These tests were conducted on a 2.60GHz Intel Xeon E5-2650 v2 CPU, and repeated using GloMPO v2.1 (which was used to produce the N-CMA optimization results) and GloMPO v3.1 (which was used for Test B).

**Table 4:** Timing tests on the Disulfide and 20D Schwefel functions showing the number of function evaluations possible within a fixed time-limit

Function	Ver.^a^	Push Freq.^b^	Function evaluations achieved^c^	GloMPO cost (ms/eval)
Disulfide$^{\mathrm{d}}$			57 ± 0	
Disulfide	v2.1	1	56 ± 0	93.98
Disulfide	v3.1	1	57 ± 0	18.53
Schwefel$^{\mathrm{d}}$			9415963 ± 032073	
Schwefel	v2.1	1	60227 ± 154	4.95
		10	516510 ± 1614	0.55
Schwefel	v3.1	1	744309 ± 30812	0.37
		10	1646672 ± 5801	0.15

Timing tests are *not* optimizations, but an infinite loop of evaluations of the same parameter vector. Functions were tested managed and unmanaged for 300 s on a single 2.60 GHz Intel Xeon E5-2650 v2 CPU. Every configuration test repeated 5 times. GloMPO cost estimates are constructed by assuming the mean unmanaged evaluation rate to be representative of the intrinsic function evaluation rate, and further assuming that the balance of the 300s evaluation time can be attributed solely GloMPO management costs

$^{\mathrm{a}}$ GloMPO version number

$^{\mathrm{b}}$ Optimizer configured to send every *n*th evaluation to the manager

$^{\mathrm{c}}$ Average and standard deviation over the 5 repeats

$$^{\mathrm{d}}$$ Unmanaged run

It is clear that the GloMPO overhead is negligible in comparison to the ReaxFF costs. For the very fast Schwefel function, however, only a fraction of the number of function evaluations can be achieved. Profiling of GloMPO v3.1 shows the performance bottleneck to be at the point where data is read off the queue into which results are fed. We do not consider the performance drop to be critical. The design intention for GloMPO was for applications with expensive and difficult functions; faster ones were tested here only for convenience.

We also note that sending evaluations to the manager periodically rather than continuously can produce a substantial speed-up. This is a practical solution in cases where the user would like to use GloMPO for management of the optimization of a fast function and does not need/want to gather a full evaluation history of the optimizers.

#### Degeneracy identification

One qualitative GloMPO advantage is its ability to explore more minima and, in this way, identify degenerate sets of solutions. We define degenerate parameter sets as those which produce similar function values but are not immediately adjacent in parameter space. This is something that a single optimizer could not do, and something that GloMPO is able to do more efficiently than sequential serial optimization. To study this effect, the Shubert function from Test A, and cobalt cost function were used. As mentioned earlier, the former has several degenerate and periodically distributed global minima. Table 5 shows the maximum, average, and total number of times answers very near the best minimum were located for the cobalt error and Shubert test functions across all bouts. This demonstrates that GloMPO is not only more likely to find the minimum at all, but also more likely to find it at different locations (if such a possibility exists).

**Table 5:** Maximum, average, and total number of degenerate parameter sets found by cobalt and Shubert functions across all bouts

Function	GloMPO			Serial		
	Max.	Sum	Mean	Max.	Sum	Mean
Cobalt$^{\mathrm{a}}$	5	31	3.100	4	18	1.800
Shubert$^{\mathrm{b}}$	4	119	0.595	3	78	0.390

Degenerates are defined as parameter sets which produce similar function values but are not immediately adjacent in parameter space

$$^{\mathrm{a}}$$ Degenerate range: $${1230}< f(x) < {1270}$$

$$^{\mathrm{b}}$$ Degenerate range: $${-39303}< f(x) < {-39000}$$

**Fig. 9:**
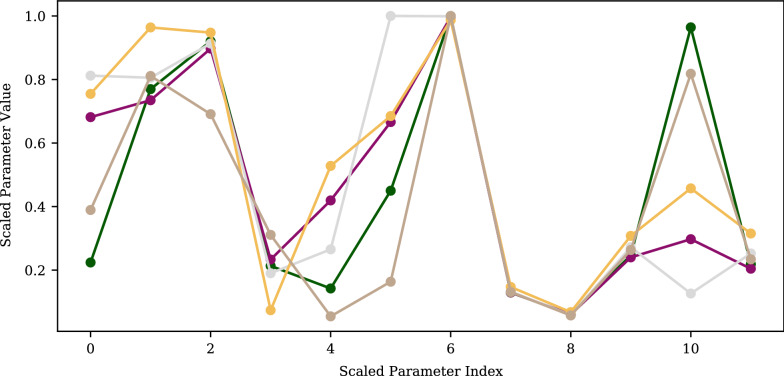
Scaled parameter set values of five cobalt parameter sets found during a single GloMPO optimization run in Set 1. (${1236}< f(x) < {1260}$)

Figure 9 shows the parameter values for five degenerate sets found in a single GloMPO optimization run in Set 1. Note, only the optimized parameters are shown, and they have been scaled between 0 and 1 for comparison. These sets produce errors ranging between 1236 to 1260. The Euclidean distances between these points range from 0.38 to 1.21. It is immediately clear that the parameter sets are correlated in some way and share many similarities.

**Fig. 10:**
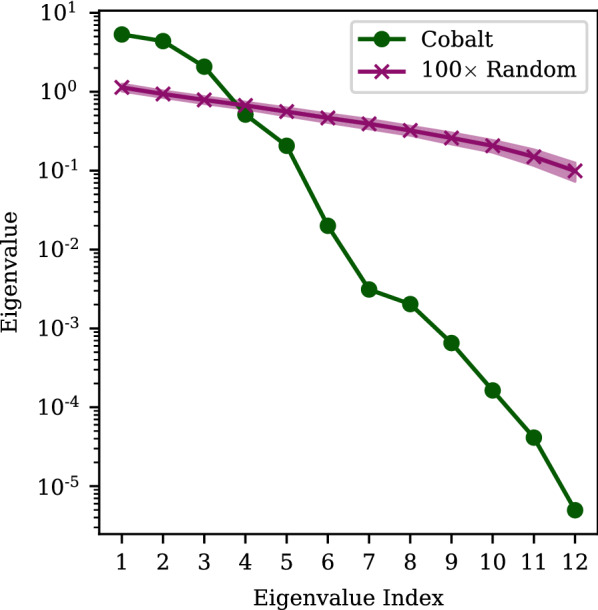
Eigenvalues of the covariance matrix of 31 degenerate cobalt parameter sets found across all GloMPO reparameterizations of this set. (1236 $< f(x)<$ 1260). For comparison, the averaged eigenvalues of the covariance matrices of 100 normally distributed randomly generated 31 $\times$ 12 matrices are also included

One can also mimic a longer GloMPO optimization by taking all 31 degenerate parameter sets found across all bouts. Figure 10 shows a PCA analysis performed on these sets. To confirm that there is not a spurious correlation, the eigenvalues for 100 sets of 31 normally distributed randomly generated vectors with 12 elements were also evaluated. The average and standard deviation of these results are show in the figure for comparison. The eigenvalues for the cobalt parameter sets clearly demonstrate a higher degree of correlation than the randomly generated parameter vectors, which tend to show some spurious correlations. It can be said that the cost function is clearly dominated by two or three dimensions; a fact which can also be seen in Fig. 9. This suggests that there may be a narrow valley of good solutions connecting these sets.

One can investigate such relationships to determine how and why they are connected. Literature support for this exists; Barcaro et al. [3] found evidence of degeneracy in their silica force field where two sets of very different parameters resulted in similar predictive results. GloMPO’s ability to better identify and group such minima may be helpful in future force field development. This can lead to a reduced number of dimensions by enforcing relationships between linked parameters. It can also reveal deficiencies in the training set. For example, parameter sets which have the same error value, but perform very differently when applied to a molecular dynamics simulation, demonstrate that the training set has not fully captured some critical property; it may also be an indication of overfitting.

#### Overfitting

We should briefly mention the problem of overfitting which is of critical importance when designing force fields for production runs. Making useful fields was not our immediate concern here, however, future authors may be interested in using validation sets in their work to guard against the problem. GloMPO handles this easily by allowing optimization tasks to return any extra data they please (like a validation set result). This information is logged, and available to the hunters. Thus, a hunter can easily be designed to terminate children which show a deterioration in the validation set. This flexibility of allowing extra information to be produced by the task, allows GloMPO to make use of any order parameter to manage the optimization. We also note that the ReaxFF interface packaged with GloMPO already includes validation set infrastructure.

#### Benchmark test results

Due to the computational expense of ReaxFF reparameterizations, only ten bouts could be carried out for these tests. Speaking in terms of win rates in such a context would be disingenuous given that a single outlier could significantly warp the results. In this section, results from each bout are presented, and GloMPO’s effect is analyzed qualitatively. For reasons that will be fully explored in “Challenges of the error function”, optimizers working on the disulfide reparameterization did not converge naturally. Each bout was stopped to limit further computational expense after 1.7 × 10^6^ total function evaluations had been used by all the optimizers combined.

**Fig. 11:**
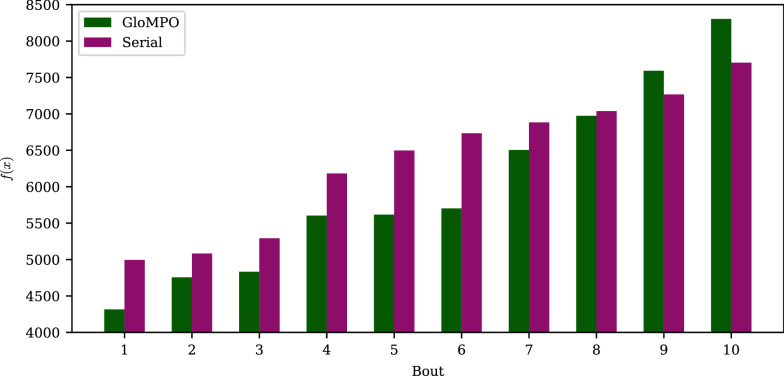
Serial and GloMPO final minima of the ReaxFF error function to reparameterize the disulfide ReaxFF force field (Set 6). Results sorted ascending

Given this fixed termination condition, bouts for the disulfide tests are not linked in the way all the other results in this work are. Figure 11 shows the minima located by the ten serial and ten GloMPO reparameterizations, each sorted in ascending order. GloMPO produced better results than serial optimizations in eight of the ten comparisons.

**Fig. 12:**
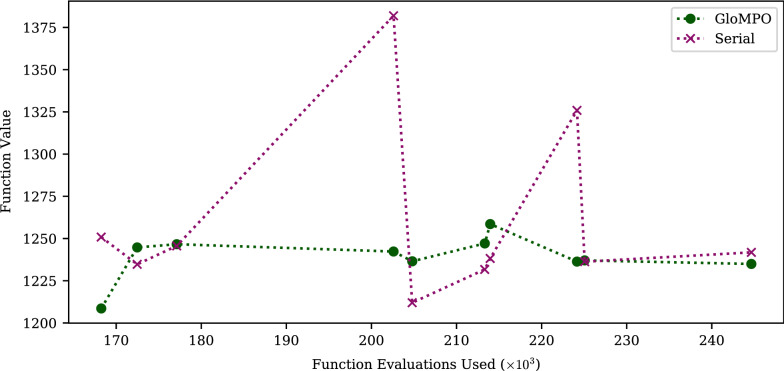
Serial and GloMPO final minima of the ReaxFF error function to reparameterize the cobalt ReaxFF force field (Set 1) as a function of number of function evaluations used

Figure 12 shows the minima generated during reparameterizations of the cobalt force field as a function of evaluations used. GloMPO consistently produces good quality answers, unlike serial which has a high variability. This behavior is also insensitive to the evaluation budget.

Broadly, GloMPO did improve the quality and quantity of the minima found during ReaxFF reparameterizations. Its performance, however, was not as decisive as its effect on Tests A and B. This can be partially attributed to ReaxFF’s particular properties which make it difficult and expensive to handle, and partially attributed to the fact that there was no information sharing or sophisticated metaheuristic used.

#### GloMPO nudging with ReaxFF

N-CMA was used on both the cobalt and disulfide error functions. Unfortunately, optimizers all converged to the minima to which they were nudged. This was usually the first minimum encountered by one of the early optimizers. This failure, however, is more a criticism of N-CMA as applied to ReaxFF, rather than of information sharing between children which can be applied in different ways, and was also shown to be important in Test B. Although these results were disappointing, investigating the reasons for this proved enlightening.

**Fig. 13:**
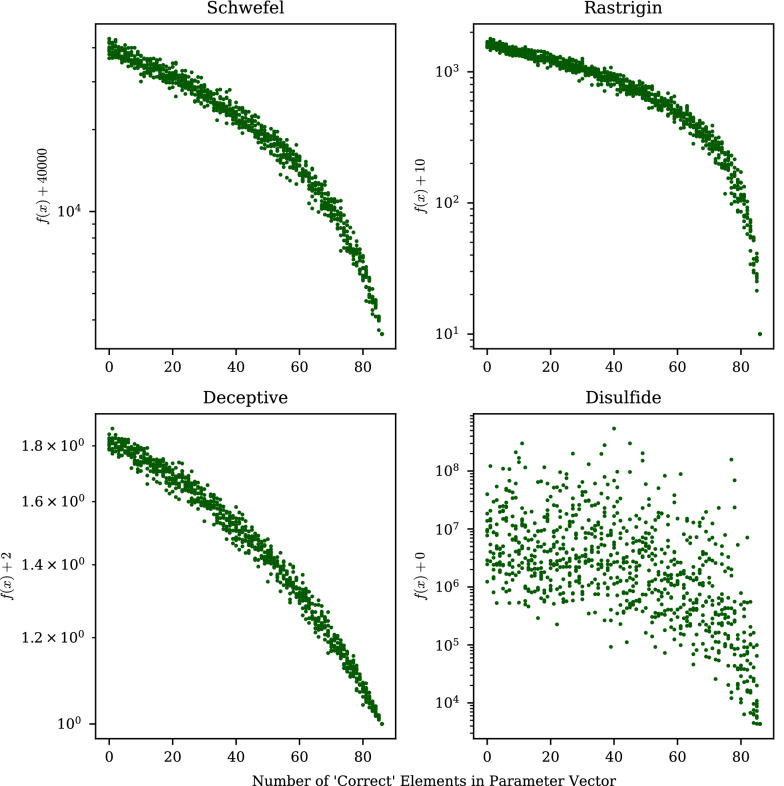
Evaluations of four optimization functions with varying number of parameters correctly set to values which produce the best minimum. Remaining parameter values are set randomly. Ten repeats are performed for each number of correctly set parameters. Some function values are shifted vertically by some amount to ensure all values are positive and all functions can be visualized comparatively on log-scales

Evaluations of the Schwefel, Deceptive, Rastrigin and disulfide error functions were studied. The disulfide error function has 87 parameters. For direct comparison, the 87-dimension versions of the mathematical test functions were used here. For each function, the best minimum was identified. In the case of the test functions, the global minimum is known. For the disulfide error function, we define the best minimum as the lowest ever found during our optimizations. For each function, 860 vectors were sampled uniformly from the domain. For ten of these, one random element in the vector was changed to the corresponding value in the best minimum vector. For another ten, two random elements were changed to the corresponding values in the best minimum vector, and so on until the final set of ten vectors containing 86 correctly set parameters and one random value. The function values corresponding to these vectors are plotted versus the number of correctly set vector elements in Fig. 13.

This figure demonstrates two things quite clearly. First, as discussed in “Optimization task”, test functions are often less complex than is sometimes assumed. Second, nudging cannot work on a function as rugged as ReaxFF. With between 10 and 20 well-set parameters, the test functions already show improvements in their function value. More than 80 parameters must be set correctly to see dramatic improvements in the disulfide error function. For the test functions, if certain parameters are correctly set, they will, on average, produce lower function values than sets with a fewer number of correctly set parameters. In other words, this can be informative for the optimizer, and encourage it to explore regions with more elements which are correctly set. This is not true for the ReaxFF error functions. The variability in function value is orders of magnitude larger than the reduction induced by setting parameters correctly. Thus, optimizers will not be able to learn what elements of the parameter vectors to replicate; nudging would be uninformative.

One can draw several important conclusions from this:

1. The ReaxFF error function can be characterized as having very many local minima, with very small basins of attraction, immediately surrounded by very high barriers. This makes locating and exploring minima extremely difficult; almost to the point of being equivalent to a random search.
2. Single parameter tuning approaches such as SOPPE can be wildly misleading, particularly without expert intervention.
3. The crossover operators in evolutionary algorithms will be limited in efficiency because they assume that a partially correct parameter vector has an evolutionary advantage, i.e., noticeably lower error. Figure 13 clearly shows this assumption barely holds for the disulfide training set.

It is unlikely that any optimization algorithm would be able to efficiently deal with such a pernicious problem. It would perhaps be more profitable to address the conditioning of the error function itself, before trying to develop optimization approaches further. One can see that an improvement trend does exist for the disulfide function in Fig. 13, but it is masked by a large amount of noise. If the error function could be better conditioned by fixing certain parameters or removing certain elements from the training set, this noise could perhaps be reduced. This would generally improve the performance of most optimization algorithms, but conceivably also unlock the potential of N-CMA.

#### Challenges of the error function

**Fig. 14:**
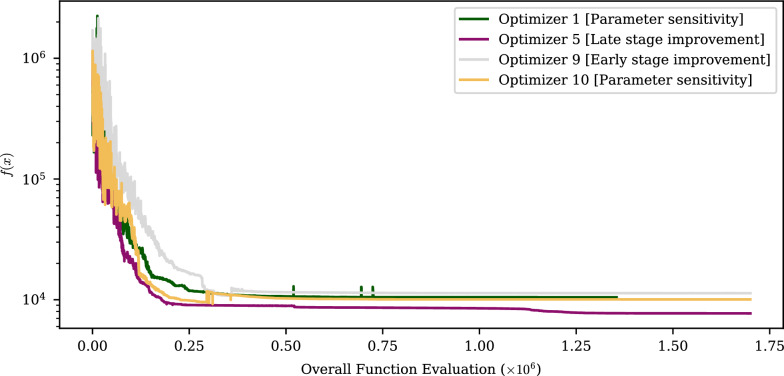
Sample of optimizer trajectories from a serial reparameterization of the disulfide ReaxFF force field. Each optimizer examples some difficulty with handling ReaxFF error functions

We close this section with some interesting insights into the behavior of the cost functions that came from analyzing the various optimization trajectories saved in the GloMPO logs. These insights can help future workers reparameterizing ReaxFF fields. Figure 14 shows an example of some of the optimizers taken from a disulfide serial optimization. Each demonstrates a difficulty when handling these types of error functions.

The first issue is high sensitivity to minute changes in parameter values. In optimizer 10, one sees the optimizer behave strangely, and oscillate between a lower and higher function value before ultimately settling at the higher one. Optimizer 1 jumps significantly several times during its long focus phase. This instability is more pronounced than it appears since only the best function evaluation is recorded per CMA iteration (17 function evaluations). In other words, to see the spike in function value, all 17 evaluations must simultaneously evaluate to the higher level, the rest of the time this behavior is happening, and effecting the optimizer’s search behavior without the user’s knowledge. The maximum difference between parameters at the lower and higher level for both optimizers is on the order of $10^{-4}$; parameters range between 0 and 1.

The parameter vectors generated by the optimizer must go through several transformations, and a loss of numerical precision in a file writing step (due to software constraints), before finally being tested in the ReaxFF model. The Python CMA-ES implementation used here also has its own internal transformations. Such high sensitivity to small parameter changes can cause spurious behaviors, as seen here. This behavior can have several serious effects: (1) it can misdirect optimizers during their exploration phase, (2) it can prevent convergence from being achieved if an optimizer is converging towards such a point, and (3) it creates the need for the user to validate the stability of the parameter set by repeated evaluations.

The second problem encountered during ReaxFF reparameterization was the late-stage improvement in function value after very long periods. The appearance of these late-stage improvements is rare but occurs frequently enough to work against GloMPO which shuts down optimizers which appear converged. A plausible explanation for this is associated with the volatility of the error function. Local information available to the optimizers is insufficient to direct them to better solutions, only when randomly sampling outside of a basin can the optimizer make improvements in the cost function value. This behavior is dependent on the type of optimizer used, CMA-ES – which randomly draws its samples from a multivariate Gaussian distribution—is susceptible to this, a deterministic algorithm, however, would not be. Another possibility is that the covariance structure of the error function changes very slowly in a given area. In this scenario, CMA would require many iterations before it has a properly updated covariance matrix and is able to sample in the correct direction. In this case, similar behavior would occur in quasi-Newton methods.

Closely linked to this phenomenon is the early-stage improvements in function value seen shortly after the optimizer appears to converge. Although occurring for the same reasons as before, early-stage improvements could be well handled by making GloMPO’s hunting conditions less strict and allowing the optimizers to remain alive for longer periods. Big improvements were seen between the disulfide bouts in Sets 5 and 6, and between the cobalt bouts in Sets 0 and 1.

**Fig. 15:**
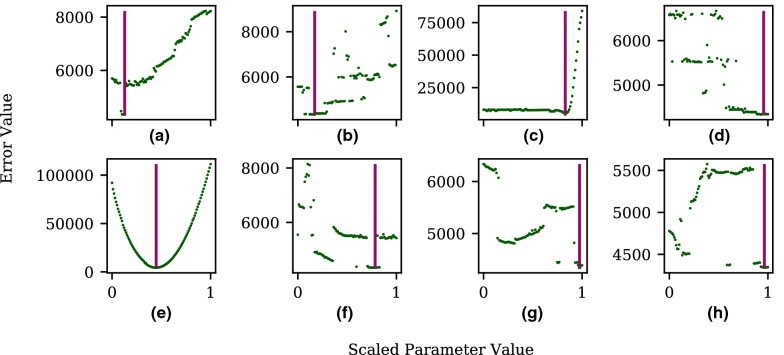
Evaluations of the ReaxFF error function for the disulfide training set against parameter values which were scanned (one at a time) from their lowest to highest bounds. Other parameters were set to the corresponding values in the reference set, i.e., the parameter set found during optimization which produced the lowest error. Of the 87 scans, 8 representative ones are presented here for brevity. The purple vertical lines show the location of the reference value for the parameter

All of the suppositions can be validated through visualizations of parameter scans of the error function. Figure 15 shows a sample of such scans performed around the parameter set which produced the lowest error for the disulfide training set. The scans were performed by evaluating the error function 100 times along each of the parameters from their lower to upper bound. Of the 87 parameters trained in the set, 8 scans are presented here. These were selected to demonstrate the different types of behavior seen while remaining representative.

Figure 15c shows an example of high sensitivity where the minimum is located very near a steep barrier, Figure 15a shows the minimum sandwiched between very steep boundaries on both sides. In either case, small changes in parameter value result in large jumps in function value. Fig. 15d, f and h show examples of the error function oscillating between two or more different function values, this can mislead the optimizers such that they oscillate between values and are unable to converge. Figure 15g is an example of how the error function can mislead the optimizer away from the minimum. Figure 15b displays little discernible trend for much of its scan. In such cases, the minimum is only found when the optimizer randomly samples a better area, thus causing the late-stage improvements seen in Fig. 14.

## Conclusions

This work introduces GloMPO (Globally Managed Parallel Optimization), a metaheuristic optimization framework, which seeks to provide a Python application through which difficult optimizations can be managed. We believe this is the first such Python framework, and the first to formalize a forced termination mechanism over a set of optimizations running in parallel. This approach is demonstrated to quantitatively improve the quality of minima found through benchmark testing on several global optimization test functions. On average GloMPO produces better results than a normal optimization given the same iteration budget. GloMPO introduces several qualitative advantages such as, providing a standardized and user-friendly interface to optimization tasks, and acting as a general workflow manager.

Further, dramatic, improvements are achieved when GloMPO is configured to share information between its managed optimizers. Similarly good performance is seen when GloMPO is configured to use basin-hopping and dual annealing algorithms through its framework on Lennard-Jones cluster problems of varying difficulty. These tests demonstrated how GloMPO can be used to mimic published metaheuristics, while offering the chance to mix and match different configurations.

GloMPO also outperforms traditional optimization when applied to ReaxFF reparameterizations. The improvements, however, are less pronounced than when applied to the mathematical test functions due to the highly oscillatory and non-robust nature of such functions. To unlock GloMPO’s demonstrated potential, work must be done to better condition ReaxFF’s error function. This involves careful study of parameter sensitivities, and the contributions within the training sets.

A further advantage GloMPO has over traditional optimization is the identification of degenerate parameter sets; parameter sets which share similar error values but differ markedly in parameter values. Such sets can help researchers identify relationships between parameters or deficiencies in the training set.

GloMPO has proven itself to be a robust framework that can aid reparameterization, and optimization efforts when computational expense or function complexity is a consideration. It certainly warrants more development. We explicitly note, however, that it is not an appropriate tool for fast functions. The use of such functions in this work was borne out of the necessity of producing a large number of results in a timely manner. In practice, however, more efficient optimization algorithms exist which have been developed in faster languages like FORTRAN or C/C++. At the opposite limit, however, where the optimizer is not the bottleneck and the researcher must carefully consider their optimization choices due to time constraints, or a pathologically misbehaving function, GloMPO is an appropriate tool to help automate those controls.

As an introductory work, we leave many aspects of the optimization management approach to be studied; we list several such examples below in the hope of stimulating further research. First, a more rigorous hunting framework should be developed, one that is able to perform well regardless of the function being studied. Second, the configurations used here were all chosen empirically. It is believed that more rigorous study of these settings could improve performance even further. Third, only the simplest selectors and generators were used in this work, but more nuanced configurations could also conceivably lead to better results. Finally, a natural extension of GloMPO seems to be the development of an analysis tool which can use the information gathered by the manager to identify problems or characteristics of the optimization task. This could be of particular use in determining how to better condition the ReaxFF error function for example.

## Supplementary Information

**Additional file 1.** 1) mathematical details of the benchmark functions, 2) algorithms for the GloMPO manager, basin-hopping and dual-annealing strategies, and 3) summaries of  optimization results.

## Data Availability

Source code availability: Project name: GloMPO. Project home page: www.github.com/mfgustavo/glompo. Operating system: Platform independent. Programming language: Python >3.6. Other requirements: > AMS2020.1 for ReaxFF interface. License: GPL-3.0. Commercial license needed for AMS2020. The dataset supporting the conclusions of this article (i.e., the raw benchmark test results) is available in the Zenodo repository, 10.5281/zenodo.5101529. Also included are the ReaxFF ffield files for the lowest error values found in each run.

## Abbreviations

AMS: Amsterdam Modeling Suite
BH: Basin-Hopping
BOF: Bijzonder Onderzoeksfonds
CMA-ES: Covariance Matrix Adaptation-Evolutionary Strategy
DA: Dual Annealing
EA: Evolutionary Algorithm
FIRE: Fast Inertial Relaxation Method
FWO: Fonds Wetenschappelijk Onderzoek
GloMPO: Globally Managed Parallel Optimization
HEB: High-dimensional, expensive and black-box
LJ: Lennard-Jones
MOF: Metaheuristic Optimization Framework
N-CMA: Nudging CMA-ES
ParAMS: Parameterization Tools for AMS
PES: Potential Energy Surface
ReaxFF: Reactive Force Field
SOPPE/I: Sequential One-Parameter Parabolic Extra/Interpolation
RiPSOGM: Rotation-invariant Particle Swarm Optimization with isotropic Gaussian Mutation
VSC: Vlaams Supercomputer Centrum
