# Supplementary material for: GloMPO (Globally Managed Parallel Optimization): a tool for expensive, black-box optimizations, application to ReaxFF reparameterizations
Source: J Cheminform. 2022 Feb 16;14:7. doi: 10.1186/s13321-022-00581-z (PMC8848815; doi:10.1186/s13321-022-00581-z)
Supplement: Supplementary file 1 — Additional file 1. 1) mathematical details of the benchmark functions, 2) algorithms for the GloMPO manager, basin-hopping and dual-annealing strategies, and 3) summaries of optimization results. [file 13321_2022_581_MOESM1_ESM.pdf]

ADDITIONAL FILE 1

GloMPO (Globally Managed Parallel Optimization) - a tool for expensive, black-box optimizations: application to ReaxFF reparameterizations

Michael Freitas Gustavo[1,2] and Toon Verstraelen[1*]

*Correspondence:
toon.verstraelen@ugent.be
[1]Center of Molecular Modeling, Ghent University, Ghent, Belgium
Full list of author information is available at the end of the article

S1 Global optimization test functions

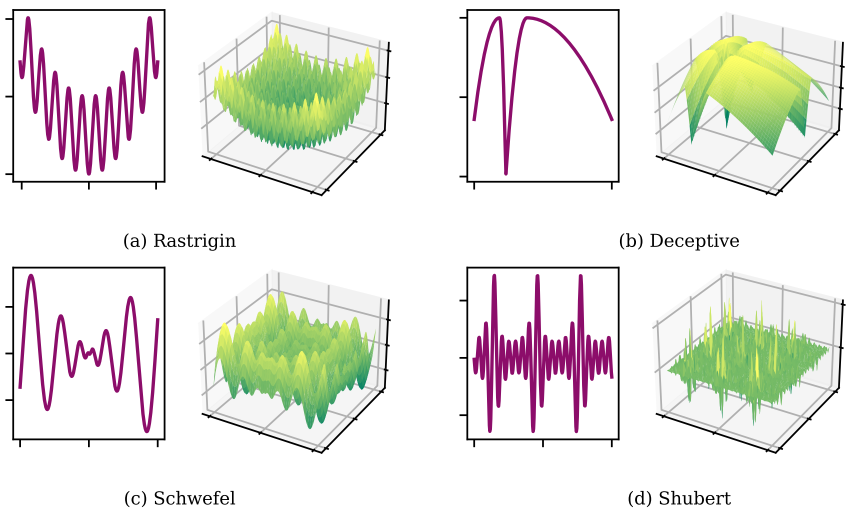

(a) Rastrigin (b) Deceptive (c) Schwefel (d) Shubert

Figure S1 One- and two-dimensional plots of the tested global optimization test functions. All plots are constructed from their respective lower to upper bounds.

Rastrigin

$$f_{\text{Rastrigin}}(\mathbf{x}) = 10d + \sum_{i=1}^{d} x_i^2 - 10\cos(2\pi x_i) \tag{S1}$$

where $d = 66$
$-5.12 < x_i < 5.12$
$\mathbf{x}_{\text{min}} = \{0, 0, \ldots, 0\}_d$
$f_{\text{Rastrigin}}(\mathbf{x}_{\text{min}}) = 0$

Source: Hoffmeister and Bäck [1]

Deceptive

$$f_{\text{Deceptive}}(\mathbf{x}) = -\left[\frac{1}{d}\sum_{i=1}^{d} g_i(x_i)\right]^{\beta} \tag{S2}$$

$$g_i(x_i) = \begin{cases} -\frac{x}{\alpha_i} + \frac{4}{5} & \text{if } 0 \le x_i < \frac{4}{5}\alpha_i \\ \frac{5x}{\alpha_i} - 4 & \text{if } \frac{4}{5}\alpha_i \le x_i < \alpha_i \\ \frac{5(x-\alpha_i)}{\alpha_i - 1} + 1 & \text{if } \alpha_i \le x_i < \frac{1+4\alpha_i}{5} \\ \frac{x-1}{1-\alpha_i} + \frac{4}{5} & \text{if } \frac{1+4\alpha_i}{5} \le x_i \le 1 \end{cases} \tag{S3}$$

where $d = 20$
$\beta = 2$
$0 < x_i < 1$
$\mathbf{x}_{\text{min}} = \alpha$
$f_{\text{Deceptive}}(\mathbf{x}_{\text{min}}) = -1$
Source: Saud and Mohamed [3]

Schwefel

$$f_{\text{Schwefel}}(\mathbf{x}) = \sum_{i=1}^{d} -x_i \sin\left(\sqrt{|x_i|}\right) \tag{S4}$$

where $d = 20$
$-500 < x_i < 500$
$\mathbf{x}_{\text{min}} = \{420.9687, 420.9687, \ldots, 420.9687\}_d$
$f_{\text{Schwefel}}(\mathbf{x}_{\text{min}}) = -418.9829d$
Source: Karaboga and Basturk [2]

Shubert

$$f_{\text{Shubert II}}(\mathbf{x}) = \prod_{j=1}^{d}\sum_{i=1}^{5} i\cos\left[(i+1)\,x_j + i\right] \tag{S5}$$

where $d = 4$
$-10 < x_i < 10$
$\mathbf{x}_{\text{min}}$ many degenerate locations
$f_{\text{Shubert}}(\mathbf{x}_{\text{min}}) = -39\,303.550\,054\,3$
Source: Wang et al [5]

S2 Management algorithm

A detailed algorithm of the GloMPO management loop, and how it uses each of the customizable classes is given in Algorithm S1. The first step of the process is to fill the available worker slots by starting new optimizers with StartNewWorkers which uses the *selector* to decide which of the available *optimizers* to start and the *generator* to select an initial starting point. Note that *optimizers* consists of optimizer types or blueprints not actual instances.

GloMPO implements two communication channels between the manager and its children. The first is a shared queue between all children. The queue is unidirectional and centralizes all the function evaluation results. This ensures that functions are collected in the order in which they are evaluated, and, by using a size-limited

queue, fast-functions can be throttled to remain in sync with the manager. The second channel is a bidirectional pipe through which the child can message the manager, and the manager can control the child. This channel ensures that an open connection is always available between the child and manager even when the queue is full. Overall, this system is more robust than using a single channel as different types of information transfer can be handled in distinct ways.

CheckOptimizerMessages polls each worker for messages about its status (i.e. checking if it has converged or crashed). This call will also perform all the necessary post-processing and cleaning-up should the worker have ended.

The manager then waits on the queue for results from the workers for a defined period of time (t). If new results are ready, these are extracted from the workers and added to the *result_log*, from which a new best solution is further extracted. This log will then be examined to determine if the *hunter* condition is satisfied for any of the optimizers. If so, such optimizers are terminated (lines 13 to 16).

At this point (line 18) the manager shares the incumbent solution with all its children. They may use this information to better guide their explorations if configured to do so.

InspectOptimizers is a routine which checks the status of the workers to ensure they have not stalled, ended without sending feedback to the manager, or failed to respond to control signals. Any transgressors are shutdown and cleaned-up. This routine is distinct from CheckOptimizerMessages since it handles irregular or unexpected behavior.

Finally, CheckConvergence determines if the *checker* condition for GloMPO termination is satisfied. If so, the loop exits, all workers are shutdown, and the best solution is returned.

S3 Mimicked metaheuristic algorithms

S3.1 Basin-hopping

Algorithm S2 is a simplified procedure for the basin-hopping algorithm as implemented in SciPy v1.2.1 [4]. Algorithm S3 details the procedure of the 'basin-hopping generator' used in Test B. The generator algorithm follows the process of the actual basin-hopping algorithm as closely as possible with some minor tweaks to allow for running local optimization in asynchronous parallel.

The optimizer routine can be broadly divided into four parts:

1 Adjust step size based on acceptance rate;
2 Randomly perturb the current location (limited by a maximum step size);
3 Run a local minimization from the new point;
4 Accept/reject the minimization's result as the starting point for new steps based on a Metropolis-Hastings test.

The generator follows the same procedures but the order is altered since its output is before point 3 i.e. it must return the new point for optimization by one of the manager's children. At first the generator returns random points until the manager has accumulated some results. Thereafter, the location from which the step is taken is the best point seen thus far by the manager from any child. This is reasonable since steps are most likely taken from the best point in the optimizer algorithm. Occationally, the step is taken from a worse point. To mimic this, the best point

Algorithm S1 Basic loop of GloMPO management structure

```
procedure MinimizeFunction(f(·), bounds)
    result ← ∞
    is_converged ← False
    workers ← empty list
    results_log ← empty list
    while not is_converged do
        workers ← workers + StartNewWorkers(f(·),
                            selector, optimizers, generator)
        CheckOptimizerMessages(workers)
        wait new_results ← GetResults(workers) timeout t
        if new_results then
            results_log ← results_log + new_results
            result ← min(results_log)
            for all worker in workers do
                kill_worker ← HuntOptimizer(hunter,
                                    results_log, worker)
                if kill_worker then
                    ShutdownWorker(worker)
                else
                    Message(worker, result)
                end if
            end for
        end if
        InspectOptimizers(workers)
        is_converged ← CheckConvergence(checker)
    end while
    for all worker in workers do
        ShutdownWorker(worker)
    end for
    LoggingAndPlotting(results_log)
    return result
end procedure
```

Algorithm S2 Simplified basin-hopping optimizer

```
procedure BHOptimizer(f(·), x_0, i_max, interval, target, Δ, factor, T)
    x ← x_0
    y ← f(x)
    n_accept ← 0
    for i in {1, 2, 3, ..., i_max} do
        if i mod interval is 0 then          ▷ Adjust step size (Δ) every interval iterations.
            accept_rate ← n_accept / i
            if accept_rate > target then
                Δ ← Δ / factor
            else
                Δ ← Δ · factor
            end if
        end if
        step ← RandomVector(−Δ, Δ)           ▷ Uniform random perturbation in each dimension.
        x_trail ← x + step
        x_trail, y_trail ← BFGS(x_0 = x_trail)   ▷ Local optimization returning new minimum.
        accept ← Metropolis(y, y_trail, T)       ▷ Metropolis-Hastings accept/reject test.
        if accept then
            x, y ← x_trail, y_trail
            n_accept ← n_accept + 1
        end if
    end for
    return x, y
end procedure
```

from another random child is chosen and accepted as the starting point with a Metropolis-Hastings test.

The generator routine can thus be summarized as follows:

1 Identify best location seen thus far;
2 Accept/reject a move to another good point explored by another child based on a Metropolis-Hastings test;
3 Adjust step size based on acceptance rate;
4 Randomly perturb the current location (limited by a maximum step size);
5 Return the new perturbed point.

Algorithm S3 Simplified basin-hopping generator

$i \leftarrow 0$
$y_{\text{old}} \leftarrow \infty$
$source_{\text{old}} \leftarrow 0$
procedure BHGenerator($manager$, $interval$, $target$, Δ, $factor$, T)
    $i \leftarrow i + 1$
    if $manager$ **is empty then** ▷ Start children in random location if no results.
        return RandomVector
    else ▷ Setup generator location as the best seen point so far.
        $x, y, source \leftarrow$ GetBestResult($manager$)
        if $y_{\text{old}} < y$ **and** $source_{\text{new}} \neq source$ **then** ▷ $source$ refers to child optimizer ID.
            $n_{\text{accept}} \leftarrow n_{\text{accept}} + 1$
        end if
    end if
    $x_{\text{other}}, y_{\text{other}} \leftarrow$ GetBestFromRandomChild($manager$)
    $accept \leftarrow$ Metropolis(y, y_{other}, T) ▷ Create chance to explore from not-best point
    if $accept$ **then**
        $x, y \leftarrow x_{\text{other}}, y_{\text{other}}$
        $n_{\text{accept}} \leftarrow n_{\text{accept}} + 1$
    end if
    if i **mod** $interval$ **is** 0 **then** ▷ Adjust step size (Δ) every $interval$ iterations.
        $accept\_rate \leftarrow n_{\text{accept}}/i$
        if $accept\_rate > target$ **then**
            $\Delta \leftarrow \Delta/factor$
        else
            $\Delta \leftarrow \Delta \cdot factor$
        end if
    end if
    $step \leftarrow$ RandomVector($-\Delta$, Δ)
    $x_{\text{trail}} \leftarrow x + step$
    return x
end procedure

This construction results in an exploration of a chain of points (as is done by the parent optimizer) but multiple steps are taken simultaneously rather than one-at-a-time. Occasionally, steps are taken from points not on the main chain to encourage exploration.

Something should be mentioned about the step-size. This is a single parameter which grows and shrinks based on the performance of the main chain, it is poorly suited to taking steps from other points when they are selected. One could develop the algorithm to have another step-size parameter for these special jumps, but we have not done so here in order to keep the generator close to its parent optimizer. If a special step results in finding a new best point, such that most new optimizers are started from there, starting a new main chain, then the step size will adapt with time to the new region.

S3.2 Dual annealing

Algorithms S4 show the simplified pseudocode for the dual annealing optimizer. In this case the algorithms match almost exactly, and our generator implementation mostly calls internal SciPy code. When the generator is called, it runs the same steps as the optimizer from lines 8 to 34. The core difference is that the generator *must* result in a location for a local search unlike the optimizer. Thus, the generator performs a check at this point to see if x is different from the last time the generator was called. If true, the new x is returned, otherwise the annealing step is repeating. The annealing can be repeated a maximum of five times before the temperature is reset and x is moved to a random location.

Algorithm S4 Simplified dual annealing optimizer

1: **procedure** DAOPTIMIZER($f(\cdot)$, $bounds$, T_0, i_{max}, q_v, q_a, $reset\_ratio$)
2: $stop \leftarrow$ False
3: $d \leftarrow$LENGTH($bounds$)
4: $i_{\text{global}} \leftarrow 0$
5: $x_{\text{visit}} \leftarrow x_{\text{best}} \leftarrow$ RANDOMVECTOR($bounds$)
6: $y_{\text{visit}}, y_{\text{best}} \leftarrow f(x_{\text{visit}})$
7: **while not** $stop$ **do**
8: **for** i **in** $\{1, 2, \ldots, i_{\text{max}}\}$ **do**
9: $T \leftarrow T0 \frac{2\exp{(q_v-1)}-1}{(i+2)\exp{(q_v-1)}-1}$ ▷ Adjust temperature based on step number.
10: **if** $i_{\text{global}} > i_{\text{max}}$ **then**
11: $stop \leftarrow$ True
12: **break**
13: **end if**
14: **if** $T/T_0 < reset\_ratio$ **then** ▷ Resets T and moves x to a random location
15: $x \leftarrow$ RANDOMVECTOR($bounds$)
16: $y \leftarrow f(x)$
17: $T \leftarrow T_0$
18: **break**
19: **end if**
20: **for** j **in** $\{1, 2, \ldots, 2d\}$ **do**
21: $x_{\text{visit}} \leftarrow$ DRAWFROMVISITDISTRIBUTION(j, x, q_v, T) ▷ First d moves change all dimensions, next d moves change dimensions one at a time.
22: $y_{\text{visit}} \leftarrow f(x_{\text{visit}})$
23: **if** $y_{\text{visit}} < y$ **then**
24: $x, y \leftarrow x_{\text{visit}}, y_{\text{visit}}$
25: **if** $y_{\text{visit}} < y_{\text{best}}$ **then**
26: $x_{\text{best}}, y_{\text{best}} \leftarrow x_{\text{visit}}, y_{\text{visit}}$
27: **end if**
28: **else**
29: $accept \leftarrow$ METROPOLIS(y_{visit}, y, q_a) ▷ Accept moves to worse locations based on a Metropolis-Hastings test.
30: **if** $accept$ **then**
31: $x, y \leftarrow x_{\text{visit}}, y_{\text{visit}}$
32: **end if**
33: **end if**
34: **end for**
35: $run\_local \leftarrow$ CHECKPERFORMANCE ▷ Local search based on various factors like the success of the annealing steps.
36: **if** run_local **then**
37: $x, y \leftarrow$ BFGS($x_0 = x$)
38: **if** $y < y_{\text{best}}$ **then**
39: $x_{\text{best}}, y_{\text{best}} \leftarrow x, y$
40: **end if**
41: **end if**
42: $stop \leftarrow$ CHECKPERFORMANCE ▷ Termination determined by performance, stagnation, number of failed improvements etc.
43: $i_{\text{global}} \leftarrow i_{\text{global}} + 1$
44: **end for**
45: **end while**
46: **return** $x_{\text{best}}, y_{\text{best}}$
47: **end procedure**

S4 Results Summaries

Table S1 Tested configurations in Test A: GloMPO benchmark tests using CMA and global optimization test functions.

Set	Optimizer		Conv.	Generator		n_s	n_g	Wins
	Serial	GloMPO	(10^x)	Serial	GloMPO			(%)
**Deceptive** $(d = 20)$								
3	CMA-ES	CMA-ES	-20	Random	Random	10	4	69
4	CMA-ES	CMA-ES	-6	Random	Random	10	4	64
**Rastrigin** $(d = 66)$								
8	CMA-ES	CMA-ES	-20	Random	Random	10	4	73
9	CMA-ES	CMA-ES	-6	Random	Random	10	4	67
**Schwefel** $(d = 20)$								
10	CMA-ES	CMA-ES	-20	Random	Random	5	4	64
11	CMA-ES	CMA-ES	-20	Random	Random	10	2	59
12	CMA-ES	CMA-ES	-20	Random	Random	10	4	65
13	CMA-ES	CMA-ES	-20	Random	Random	10	7	67
14	CMA-ES	CMA-ES	-20	Random	Random	10	10	63
15	CMA-ES	CMA-ES	-20	Random	Random	15	4	64
16	CMA-ES	CMA-ES	-20	Random	Random	20	4	71
17	CMA-ES	CMA-ES	-11	Random	Random	5	4	57
18	CMA-ES	CMA-ES	-11	Random	Random	10	2	63
19	CMA-ES	CMA-ES	-11	Random	Random	10	4	64
20	CMA-ES	CMA-ES	-11	Random	Random	10	7	61
21	CMA-ES	CMA-ES	-11	Random	Random	10	10	64
22	CMA-ES	CMA-ES	-11	Random	Random	15	4	71
23	CMA-ES	CMA-ES	-11	Random	Random	20	4	54
24	CMA-ES	CMA-ES	-6	Random	Random	5	4	60
25	CMA-ES	CMA-ES	-6	Random	Random	10	2	58
26	CMA-ES	CMA-ES	-6	Random	Random	10	4	67
27	CMA-ES	CMA-ES	-6	Random	Random	10	7	65
28	CMA-ES	CMA-ES	-6	Random	Random	10	10	62
29	CMA-ES	CMA-ES	-6	Random	Random	15	4	62
30	CMA-ES	CMA-ES	-6	Random	Random	20	4	53
31	CMA-ES	N-CMA	-11	Random	Incumbent	5	4	85
32	CMA-ES	N-CMA	-11	Random	Incumbent	10	2	88
33	CMA-ES	N-CMA	-11	Random	Incumbent	10	4	80
34	CMA-ES	N-CMA	-11	Random	Incumbent	10	7	91
35	CMA-ES	N-CMA	-11	Random	Incumbent	10	10	91
36	CMA-ES	N-CMA	-11	Random	Incumbent	15	4	89
37	CMA-ES	N-CMA	-11	Random	Incumbent	20	4	86
38	CMA-ES	N-CMA	-11	Random	Random	5	4	83
39	CMA-ES	N-CMA	-11	Random	Random	10	2	70
40	CMA-ES	N-CMA	-11	Random	Random	10	4	86
41	CMA-ES	N-CMA	-11	Random	Random	10	7	92
42	CMA-ES	N-CMA	-11	Random	Random	10	10	93
43	CMA-ES	N-CMA	-11	Random	Random	15	4	87
44	CMA-ES	N-CMA	-11	Random	Random	20	4	82
45	N-CMA	N-CMA	-11	Incumbent	Incumbent	5	4	70
46	N-CMA	N-CMA	-11	Incumbent	Incumbent	10	2	57
47	N-CMA	N-CMA	-11	Incumbent	Incumbent	10	4	72
48	N-CMA	N-CMA	-11	Incumbent	Incumbent	10	7	76
49	N-CMA	N-CMA	-11	Incumbent	Incumbent	10	10	77
50	N-CMA	N-CMA	-11	Incumbent	Incumbent	15	4	66
51	N-CMA	N-CMA	-11	Incumbent	Incumbent	20	4	66
**Shubert** $(d = 4)$								
52	CMA-ES	CMA-ES	-20	Random	Random	10	4	72
53	CMA-ES	CMA-ES	-6	Random	Random	10	4	54

- 'Conv.', n_s and n_g refer to the convergence settings, number of serial optimizers, and number of GloMPO optimizers used respectively (see Table 2).
- 100 repeats were run for every set.

Table S2 Tested configurations in Test B: GloMPO benchmark tests mimicking dual annealing and basin-hopping strategies on the Lennard-Jones cluster problem.

Set	Optimizer		Generator		n_s	n_g	Wins
	Serial	GloMPO	Serial	GloMPO			(%)
**Lennard Jones 10 ($d = 30$)**							
100	Dual Annealing	BFGS	Random	Dual Annealing	1	4	99
108	Dual Annealing	BFGS	Random	Dual Annealing	4	4	98
104	Basin-Hopping	BFGS	Random	Basin-Hopping	1	4	99
110	Basin-Hopping	BFGS	Random	Basin-Hopping	4	4	99
**Lennard Jones 25 ($d = 75$)**							
101	Dual Annealing	BFGS	Random	Dual Annealing	1	4	97
109	Dual Annealing	BFGS	Random	Dual Annealing	4	4	97
105	Basin-Hopping	BFGS	Random	Basin-Hopping	1	4	91
111	Basin-Hopping	BFGS	Random	Basin-Hopping	4	4	89
**Lennard Jones 50 ($d = 150$)**							
102	Dual Annealing	BFGS	Random	Dual Annealing	1	4	97
106	Basin-Hopping	BFGS	Random	Basin-Hopping	1	4	88
**Lennard Jones 75 ($d = 150$)**							
103	Dual Annealing	BFGS	Random	Dual Annealing	1	4	100
107	Basin-Hopping	BFGS	Random	Basin-Hopping	1	4	86

- n_s and n_g refer to the number of serial and GloMPO optimizers used respectively (see Table 2).
- 100 repeats were run for every set. The local optimizers had a convergence tolerance of 10^{-5}.

Table S3 Tested configurations in Test C: GloMPO benchmark tests on the reparameterization of ReaxFF force fields.

Set	Optimizer		Conv.	Hunting	Generator		n_s	n_g	Wins
	Serial	GloMPO	(10^x)	Config.	Serial	GloMPO			(%)
**Cobalt ($d = 12$)**									
0	CMA-ES	CMA-ES	-6	Strict	Random	Random	10	4	30
1	CMA-ES	CMA-ES	-6	Loose	Random	Random	10	4	40
2	CMA-ES	N-CMA	-6	Loose	Random	Incumbent	10	4	30
**Disulfide ($d = 87$)**									
5	CMA-ES	CMA-ES	-5	Strict	Random	Random	10	4	30
6	CMA-ES	CMA-ES	-5	Loose	Random	Random	10	4	50
7	CMA-ES	N-CMA	-5	Loose	Random	Incumbent	10	4	10

- 'Conv.', n_s and n_g refer to the convergence settings, number of serial optimizers, and number of GloMPO optimizers used respectively (see Table 2).
- 10 repeats were run for every set.
- 'Loose' and 'Strict' hunting configurations refer to how aggressively GloMPO shutdown child optimizers. A 'Loose' hunting style allowed optimizer to remain alive for longer, well into the focus phase. 'Strict' hunting terminated the optimizers as soon as they began to appear to focus.

Author details
[1]Center of Molecular Modeling, Ghent University, Ghent, Belgium. [2]Software for Chemistry and Materials, De Boelelaan 1083, 1081 HV, Amsterdam, The Netherlands.

References

1. Hoffmeister F, Bäck T (1991) Genetic algorithms and evolution strategies: similarities and differences. In: Lecture Notes in Computer Science (including subseries Lecture Notes in Artificial Intelligence and Lecture Notes in Bioinformatics), Springer Verlag, vol 496 LNCS, pp 455–469, URL https://doi.org/10.1007/BFb0029787
2. Karaboga D, Basturk B (2007) A powerful and efficient algorithm for numerical function optimization: artificial bee colony (ABC) algorithm. Journal of Global Optimization 39(3):459–471, URL https://doi.org/10.1007/s10898-007-9149-x
3. Saud LJ, Mohamed MJ (2014) Investigating the guidance feature of searching in the genetic algorithm. Iraqi Journal of Computers, Communication, Control & Systems Engineering 14(1):21–34
4. Virtanen P, Gommers R, Oliphant TE, Haberland M, Reddy T, Cournapeau D, Burovski E, Peterson P, Weckesser W, Bright J, van der Walt SJ, Brett M, Wilson J, Millman KJ, Mayorov N, Nelson ARJ, Jones E, Kern R, Larson E, Carey CJ, Polat I, Feng Y, Moore EW, VanderPlas J, Laxalde D, Perktold J, Cimrman R, Henriksen I, Quintero EA, Harris CR, Archibald AM, Ribeiro AH, Pedregosa F, van Mulbregt P, SciPy v1 Contributors (2020) SciPy 1.0: fundamental algorithms for scientific computing in Python. Nature Methods 17:261–272, URL https://doi.org/10.1038/s41592-019-0686-2
5. Wang X, Wang S, Xiao L (2010) Solving Shubert function optimization problem by using thermodynamics evolutionary algorithm. In: 2010 International Conference on Biomedical Engineering and Computer Science, pp 1–4, URL https://doi.org/10.1109/ICBECS.2010.5462350
